# Supplementary figures and images for: Comparison of growth models to describe growth from birth to 6 years in a Beninese cohort of children with repeated measurements
Source: BMJ Open. 2020 Sep 18;10(9):e035785. doi: 10.1136/bmjopen-2019-035785 (PMC7511607; doi:10.1136/bmjopen-2019-035785)

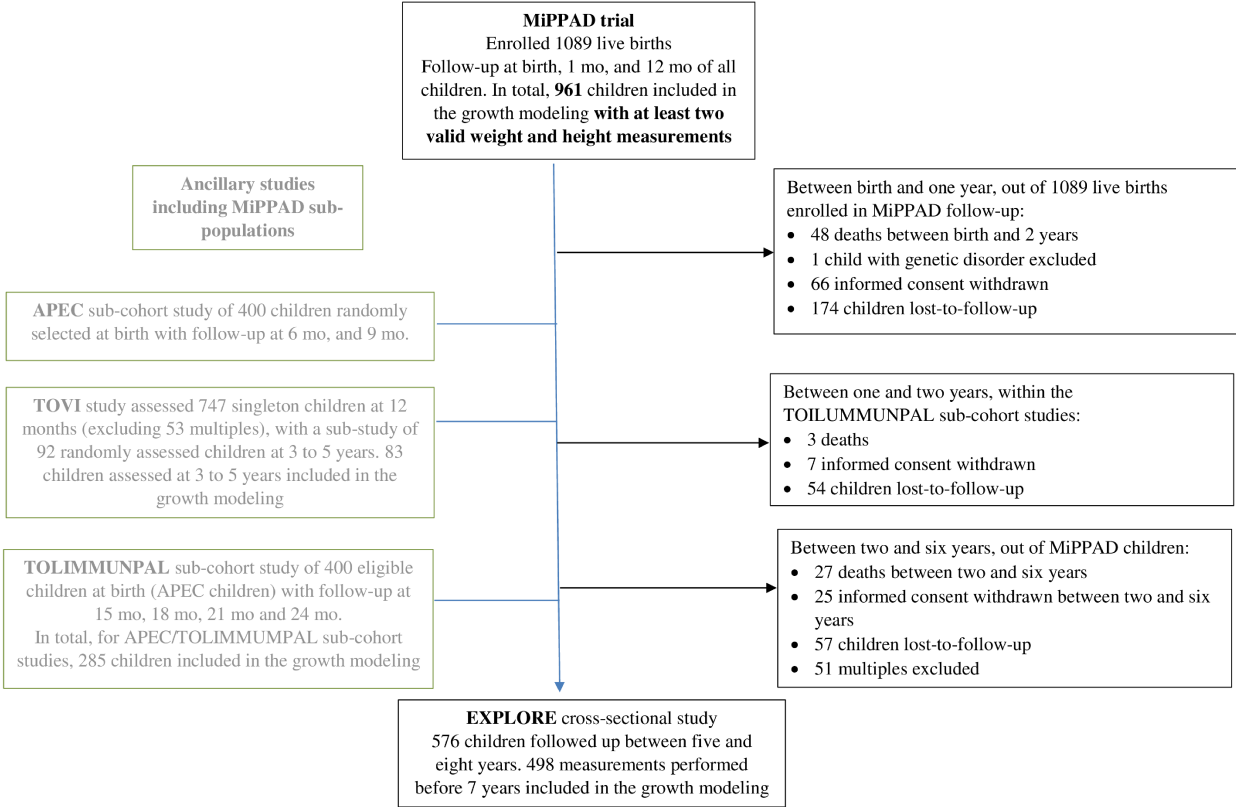

Supplement: Supplementary data [file bmjopen-2019-035785supp002.pdf]
