# Supplementary material for: Comparison of growth models to describe growth from birth to 6 years in a Beninese cohort of children with repeated measurements
Source: BMJ Open. 2020 Sep 18;10(9):e035785. doi: 10.1136/bmjopen-2019-035785 (PMC7511607; doi:10.1136/bmjopen-2019-035785)
Supplement: Supplementary data [file bmjopen-2019-035785supp001.pdf]

Supplementary tables

Supplementary Table 1. Number of children with anthropometric measurements at different age intervals. Data from boys (n=461) and girls (n=500) from birth to around six years of age

Age group	Boys	Girls
	n	n
0 – 1 yr	461	499
1 - 2 yr	196	219
2 - 3 yr	97	115
3 - 4 yr	8	10
4 - 5 yr	41	24
5 – 7 yr	253	249

Supplementary Table 2. Summary of maternal and child characteristics in the prospective child cohort in Benin, Sub-Saharan Africa.

	**Boys (n= 461)**		**Girls (n=500)**	
	**n/N**	**%**	**n/N**	**%**
Center				
Allada	51/371	13.8	49/400	12.3
Attogon	83/371	22.4	104/400	26.0
Sékou	237/371	62.9	247/400	61.8
Maternal characteristics				
Mean maternal age at the start of follow up (yr)	451/461	26.0 +/- 5.4	490/500	25.6 +/- 5.5
Maternal education [a]	112/451	24.8	106/490	21.6

Ahmadi S, *et al. BMJ Open* 2020; 10:e035785. doi: 10.1136/bmjopen-2019-035785

**Supplementary table 2. continued**				
Estimated maternal pre-pregnancy BMI (kg/m^2)				
Underweight (<18.5)	61/373	16.4	67/400	16.8
Normal (18.5 – 24.9)	262/373	70.2	298/400	74.5
Overweight (25-29.9)	35/373	9.4	26/400	6.5
Obese (≥ 30)	15/373	4.02	9/400	2.3
Gravidity				
Multigravida	305/373	81.8	326/400	81.5
Primigravida	68/373	18.2	74/400	18.5
Child characteristics				
Low birth weight (<2.5 kg)	39/440	8.9	66/481	13.7
Preterm	28/443	6.3	33/484	6.8
Malaria at 1 year	35/351	10.0	38/348	10.9
Anemia at 1 year (Hb≤110 g/L)	261/354	73.7	233/348	66.9

[a] Defined as the ability to read or write

Supplementary Table 3. Parameters estimates for three candidate models [a] fitted to weight and height

Model	Parameters	Boys		Girls	
		Mean	SD	Mean	SD
Weight models					
Jenss					
	A	1.11	0.05	1.08	0.06
	B	5.53	0.15	5.55	0.18
	C	1.52	0.16	1.49	0.15
	D	4.97	0.24	5.15	0.37
Adapted Gompertz	A	2.02	0.08	1.96	0.07
	B	0.90	<0.01	0.85	< 0.01
	C	4.67	0.24	4.85	0.25
	D	5.50	0.13	5.49	0.15
Reed	A	-1.18	<0.01	-0.89	0.12
	B	-5.87	0.18	-5.78	0.18
	C	0.39	0.08	0.28	0.07
	D	1.44	<0.01	1.34	<0.01
Height models					
Jenss					
	A	3.90	0.02	3.90	0.02
	B	3.90	0.08	3.90	0.06
	C	2.90	0.01	2.90	0.06
	D	4.90	0.09	5.00	0.07

Supplementary table 3. continued

Adapted Gompertz	A	4.21	0.02	4.2	0.02
	B	0.31	< 0.01	0.3	<0.01
	C	4.83	0.13	4.9	0.12
	D	3.92	0.07	3.9	0.06
Reed	A	3.50	0.03	3.5	0.03
	B	-4.20	0.09	-4.2	0.08
	C	1.80	0.02	1.7	0.02
	D	2.80	< 0.01	2.8	<0.01

[a] the sign of the parameters for the adapted Gompertz and the Reed model was constrained by using exponential functions, as shown in equation 2 for the Jenss-Bayley model.

Supplementary Table 4. Stunting, underweight and wasting in children using predicted data from the Jenss-Bayley model. n (%)

Sex	Underweight (WAZ<–2)	Stunting (HAZ<-2)	Wasting (WHZ<-2)
Boys (n=461)	161/461 (35)	37/461 (8)	125/451 (27.7)
Girls (n=500)	168/500 (34)	22/500 (4)	190/495 (38.4)

Ahmadi S, *et al. BMJ Open* 2020; 10:e035785. doi: 10.1136/bmjopen-2019-035785
