# Supplementary material for: Comparison of growth models to describe growth from birth to 6 years in a Beninese cohort of children with repeated measurements
Source: BMJ Open. 2020 Sep 18;10(9):e035785. doi: 10.1136/bmjopen-2019-035785 (PMC7511607; doi:10.1136/bmjopen-2019-035785)
Supplement: Author's manuscript [file bmjopen-2019-035785.draft_revisions.pdf]

BMJ Open

BMJ Open is committed to open peer review. As part of this commitment we make the peer review history of every article we publish publicly available.

When an article is published we post the peer reviewers' comments and the authors' responses online. We also post the versions of the paper that were used during peer review. These are the versions that the peer review comments apply to.

The versions of the paper that follow are the versions that were submitted during the peer review process. They are not the versions of record or the final published versions. They should not be cited or distributed as the published version of this manuscript.

BMJ Open is an open access journal and the full, final, typeset and author-corrected version of record of the manuscript is available on our site with no access controls, subscription charges or pay-per-view fees (http://bmjopen.bmj.com).

If you have any questions on BMJ Open's open peer review process please email info.bmjopen@bmj.com

BMJ Open

Comparison of Growth Models to Describe Growth from birth to 6 years in Children with repeated measurements

Journal:	*BMJ Open*
Manuscript ID	bmjopen-2019-035785
Article Type:	Original research
Date Submitted by the Author:	15-Nov-2019
Complete List of Authors:	AHMADI, Shukrullah; INSERM, Centre of Research in Epidemiology and Statistics/CRESS ; Université de Paris,<br>Bodeau-Livinec, Florence; EHESP; INSERM, Centre of Research in Epidemiology and Statistics/CRESS<br>Zoumenou, Roméo ; Institut de Recherche pour le Développement (IRD), Université Paris Descartes, MERIT (Mère et Enfant Face aux Infections Tropicales)-UMR 216<br>Garcia, Andre; Institut de Recherche pour le Développement (IRD), Université Paris Descartes, MERIT (Mère et Enfant Face aux Infections Tropicales)-UMR 216<br>Courtin, David; Institut de Recherche pour le Développement (IRD), Université Paris Descartes, MERIT (Mère et Enfant Face aux Infections Tropicales)-UMR 216<br>Alao, Jules; Mother and Child University and Hospital Center (CHU-MEL), Paediatric Department<br>Fievet, Nadine ; Institut de Recherche pour le Développement (IRD), Université Paris Descartes, MERIT (Mère et Enfant Face aux Infections Tropicales)-UMR 216<br>Cot, Michel; Institut de Recherche pour le Développement (IRD), Université Paris Descartes, MERIT (Mère et Enfant Face aux Infections Tropicales)-UMR 216<br>Massougbodji, Achille; Université d'Abomey-Calavi Faculté des Sciences de la Santé<br>Botton, Jérémie ; Agence nationale de sécurité du médicament et des produits de santé, EPI-PHARE, Epidemiology of health products
Keywords:	STATISTICS & RESEARCH METHODS, EPIDEMIOLOGY, PAEDIATRICS

SCHOLARONE™
Manuscripts

I, the Submitting Author has the right to grant and does grant on behalf of all authors of the Work (as defined in the below author licence), an exclusive licence and/or a non-exclusive licence for contributions from authors who are: i) UK Crown employees; ii) where BMJ has agreed a CC-BY licence shall apply, and/or iii) in accordance with the terms applicable for US Federal Government officers or employees acting as part of their official duties; on a worldwide, perpetual, irrevocable, royalty-free basis to BMJ Publishing Group Ltd ("BMJ") its licensees and where the relevant Journal is co-owned by BMJ to the co-owners of the Journal, to publish the Work in this journal and any other BMJ products and to exploit all rights, as set out in our licence.

The Submitting Author accepts and understands that any supply made under these terms is made by BMJ to the Submitting Author unless you are acting as an employee on behalf of your employer or a postgraduate student of an affiliated institution which is paying any applicable article publishing charge ("APC") for Open Access articles. Where the Submitting Author wishes to make the Work available on an Open Access basis (and intends to pay the relevant APC), the terms of reuse of such Open Access shall be governed by a Creative Commons licence – details of these licences and which Creative Commons licence will apply to this Work are set out in our licence referred to above.

Other than as permitted in any relevant BMJ Author's Self Archiving Policies, I confirm this Work has not been accepted for publication elsewhere, is not being considered for publication elsewhere and does not duplicate material already published. I confirm all authors consent to publication of this Work and authorise the granting of this licence.

For peer review only

Comparison of Growth Models to Describe Growth from birth to 6 years in Children with repeated measurements

Ahmadi S [1, 2], Bodeau-Livinec F [1,3] , Zoumenou R[4], Garcia A[4], Courtin D[4], Maroufou JA[5], Fievet N[4], Cot M[4,], Massougbodji A[6], Botton J[7].

[1] Obstetrical, Perinatal, and Pediatric Epidemiology Team, Centre of Research in Epidemiology and Statistics Sorbonne Paris Cité (CRESS UMR 1153), Inserm, Paris, France. shukrullah.ahmadi@inserm.fr.
[2] Paris Descartes University, Paris, France.
[3] EHESP, F-35000 Rennes, France. florence.bodeau-livinec@ehesp.fr
[4] MERIT (Mère et Enfant Face aux Infections Tropicales)-UMR 216, Institut de Recherche pour le Développement (IRD), Université Paris Descartes, 75006 Paris, France. zoumenour@yahoo.fr; andre.garcia@ird.fr; david.courtin@ird.fr; nadine.fievet@ird.fr; michel.cot@ird.fr
[5] Paediatric Department, Mother and Child University and Hospital Center (CHU-MEL), Cotonou, Benin. amomj@yahoo.fr
[6] Faculté des Sciences de la Santé, Université d'Abomey-Calavi, Cotonou, Benin. massougbodjiachille@yahoo.fr
[7] EPI-PHARE, Epidemiology of health products, Saint-Denis, France. jeremie.botton@ansm.sante.fr

Correspondence to Shukrullah Ahmadi, Obstetrical, Perinatal, and Pediatric Epidemiology Team. Centre of Research in Epidemiology and Statistics Sorbonne Paris Cité. Inserm. 4, rue de la Chine, Paris 75020. shukrullah.ahmadi@inserm.fr

Word count : 2786

For peer review only

ABSTRACT

Objective The aim of this paper was to find a growth model that describes at best individual growth trajectories of children from birth to six years in a low-income country in Sub-Saharan Africa and to show its usefulness in describing individual growth.

Design This study compared the fit of three growth models (i.e. Jenss-Bayley, Reed and a new model which is an adapted version of the Gompertz model) on longitudinal weight and height growth data of boys and girls between birth and six years from a prospective cohort in Benin, West Africa. The goodness-of-fit of the models was compared mainly using Akaike Information Criterion (AIC) and Bayesian Information Criterion (BIC). The best model allowed to predict individual weight and height growth of children.

Results The Jenss-Bayley model had the best fit for the weight in boys (e.g. Jenss vs. Reed vs. adapted Gompertz model, AIC: 5251 vs. 5387 vs. 5368) and girls (AIC: 5452 vs. 5581 vs. 5587, respectively). The Jenss-Bayley model had also the closest fit for the height in boys (AIC: 11786 vs. 11794 vs. 11792, respectively) and girls (AIC: 12711 vs. 12739 vs. 12743, respectively). 35, 8 and 27% of boys and 34, 8, and 27% of girls were found to be underweight, stunted, and wasted, respectively.

Conclusion The Jenss-Bayley model presented the closest fit to weight and height growth data from a semi-rural African setting. The growth parameters of this model can be used to describe growth trajectories and study their determinants.

Keywords Weight, height, Growth modelling, Mixed effect models, Structural models, Africa

For peer review only

Strengths and limitations of this study

- This is the first study to assess the comparative merits of growth models on child anthropometric data from birth to six years of age in a semi-rural setting in Sub-Saharan Africa with multiple repeated measures.
- A new model which is an adaptation of the Gompertz model has been evaluated to test whether it shows a better fit compared to growth models previously described.
- We can predict weight and height for all children at any age points even when not measured; and we showed its usefulness in calculating mean and individual growth trajectories and velocities, and in assessing the extent of undernutrition (underweight, stunting and wasting).
- Although we showed that the model is useful to assess the prevalence of undernutrition the generalizability of these results cannot be guaranteed because the study sample is not representative of the Beninese children: these children lived in a semi-rural setting with potentially different characteristics and risk factors of growth so it is possible that their growth would differ from urban children. For instance, this population presented a high prevalence of infections (for e.g. malarial and helminths), micronutrient deficiency (including anemia), and elevated blood lead levels[1-3].
- Although longitudinal studies are crucial in monitoring child growth, modelling longitudinal growth data is often challenging. We faced minor issues in modelling unstructured variance-covariance matrix of the random effects using SAEMIX package. In spite of this issue, the fit of the models was very optimal.

For peer review only

INTRODUCTION

Insufficient growth during childhood adversely affects later health outcomes. According to estimates from the Global Burden of Diseases, Injuries, and Risk Factors Study 2016[4], the prevalence of stunting, wasting, and underweight (based on the definitions using the WHO 2006 growth standards) in children under five were, respectively, 36.6%, 8.6% and 19.5% in sub-Saharan Africa (SSA) in 2015. Furthermore, more than 23% of under-five mortality was attributable to child growth failure, being thus the second leading risk factor for child mortality in this region. The urban–rural disparities in child malnutrition in developing countries is also widespread[5].

Mathematical growth modelling consists of fitting models to physical growth data with the aim of obtaining an appropriate growth curve that will conveniently summarize growth information[6]. Many authors have used several growth models to fit a growth curve to human weight and height measurements, amongst them the Jenss-Bayley model[7], the Reed model[8], and the Gompertz functions[9, 10].

Many studies[7, 11-27] have analyzed child growth data in low- and middle-income countries, but very few have collected data longitudinally[28]. In addition, few studies[7, 8, 12, 20, 28-30] have applied structural or non-structural growth models on African longitudinal growth data[28]. However, the comparative merits of these models have not been assessed.

This study aimed to compare the fit of three growth models (the Jenss-Bayley model, the Reed model, and a new model which is an adaptation of the Gompertz model) applied to weight and height growth data in a population of Beninese children between birth and 6 years of age, living in a semi-rural region and to describe their growth using the selected model.

For peer review only

METHODOLOGY

Study design and data sources

We used weight and height measurements from a prospective cohort study of Beninese children followed from birth to six years of age. These children participated in different follow-up studies, for which research objectives, design, sample size, and follow-up period varied.

Participating children were born of HIV-negative mothers (n = 1182) enrolled in a multi-country clinical trial (NCT00811421) in 2011 in Benin in the MiPPAD (Malaria in Pregnancy Preventive Alternative Drugs) study[31]. Participants were enrolled in two health centers, Attogon and Sékou, in the district of Allada, a semi-rural region located in the South of Benin. Height and weight of children were assessed at birth, 1-2 month, 9 months, and 12 months in the MiPPAD study.

Within this cohort, a sub-sample of 400 children were followed more closely. These children were followed from birth to two years of age in the APEC (Anemia in pregnancy: etiology and consequences)[32] and TOLIMMUNPAL studies[33]. Anthropometric measurements were completed between birth and 24 months. Later, height and weight were reassessed for a sub-sample of children (n= 92) between 3 and 5 years of age within the TOVI study. Finally, at 6 years of age, growth was measured in a follow-up study, the EXPLORE (2016 -2018)[34, 35].

Patient and public involvement

Patients and the public were not directly involved in the planning of this study. Results will be communicated to participants via community meetings with local leaders.

Anthropometric measurement

Measurements were performed by trained staff at each visit, with children wearing light clothes only and no shoes. From birth up to around 2 years, infants' length was measured to the

For peer review only

nearest 1 mm using a locally manufactured wooden measuring scale according to the WHO guidelines[36]. From birth up to around 2 years, infants' weight was measured using an electronic baby scale (SECA type 354) with a precision of 10 g. From around 2 years onwards standing weight and height was measured. Height was measured by a locally manufactured length board. The length board comprised of a measuring board mounted to the wall with an attached measuring tape and moveable right angle headpiece. Weight was measured using an analog scale (CAMRY DT602). At six years, children were weighted with the Tanita scale.

Data inclusion

Participants with at least two weight or height measurements anytime between birth and around six years of age were included in the analysis. Weight-for-age z-scores (WAZ) and height-for-age z-scores (HAZ) were calculated based on the WHO growth standards[37]. In order to identify outliers, as the distribution of the Z-scores was shifted to the left, we corrected it by centering it to the mean. Then measurements with centered WAZ or HAZ greater than +4 or less than -4 were considered outliers for growth and were systematically excluded from the analysis. Additionally, individual weight and height profiles were plotted to visually check individual growth trajectories for plausibility. The final analyses had 961 participants (461 boys and 500 girls).

Growth Models

The following three structural growth models were compared: the Jenss-Bayley model, the Reed model, and a new model which is an adaptation of the Gompertz model. These models are presented in the equations 1, 4 and 5, where E (y_{ij}) is the expected value (weight or length of child i at j^{th} occasion, t_{ij} is age in days, A_i, B_i C_i and D_i are the parameters of the functions for the ith child.

The Jenss-Bayley model [38] is presented in equation 1 and as another parameterization in equation 2. Briefly, a or $\exp^{A}$ is the predicted value at birth (t=0); b or $\exp^{-B}$ is the

For peer review only

asymptomatic slope or growth rate from about two years onwards; d or exp^{-D} allows for decelerating growth (decreasing exponential function) during infancy. The parameter c or exp^{C} can reflect the degree of catch-up growth. These four parameters or model coefficients can be obtained for each individual-thanks to a mixed effect modelling approach- and represent the global individual growth trajectory. Parameters (or a combination of them, e.g. the velocity) can then be studied in association with the determinants of growth or other independent variables[39].

$$E(y_{ij}) = a_i + b_i \cdot t_{ij} - (\exp^{c_i + d_i \cdot t_{ij}}) \quad (1)$$

$$E(y_{ij}) = exp^{A_i} + exp^{-B_i} \cdot t_{ij} + exp^{C_i} \cdot (1 - exp^{-exp^{-D_i} \cdot tij}) \quad (2)$$

The Gompertz model (equation 3 with Di=0) is a 3-parameter model commonly used to model human growth and development[40, 41]. Parameter A_i represents the upper asymptote (maximum value), C_i represents growth rate and B_i is related to weight at birth (t=0). The Gompertz model theoretically approaches a null asymptote at later ages and to better represent the positive linear growth between about 3 and 6 years of age, we expanded the Gompertz growth function[41, 42] by adding a 4th parameter D_i on height and weight modelling. The model is non-linear in the model parameters.

$$E(y_{ij}) = A_i * exp(-B_i * exp(-C_i * t_j)) + D_i * t_{ij} \quad (3)$$

In the Reed model[43], parameter B is the asymptotic slope or growth rate/velocity of preschool growth, C is an important component of rapid early childhood growth[6, 44] and models the exponential growth deceleration, D is used to better model early life variations in growth, and A is the intercept. The model is as follows:

$$E(y_{ij}) = A_i + B.t_{ij} + C_i.\ln(t_{ij} + 1) + \frac{D_i}{t_{ij}} \quad (5)$$

Statistical methods

The Jenss-Bayley model, the Reed model, and the adapted Gompertz model were applied to weight and height growth data of children from birth to 6 years of age.

We first fit the Reed fixed-effects model using the lm function, and the Jenss and Gompertz models using the nls function to obtain initial values more easily[39]. Then the models were fitted to weight and height data using the SAEMIX[45] package in R-3.5.1 software, as applied in another study[39]. Due to convergence issues with unstructured matrices, a diagonal variance-covariance matrix was selected for random effects.

Growth trajectories of the children were estimated using a recently proposed method described elsewhere[39]. Briefly, weight and height growth trajectories and growth velocities (first derivative of the equation) were estimated using the model presenting the best fit. A set of parameters obtained from the mixed-effects model were used to represent average growth trajectories. The best performing model then allowed predicting weight and height by substituting the model parameters into their corresponding model equations. Similarly, growth velocities were calculated by substituting determined individual parameters into the derivative of its equation.

Finally, undernutrition (underweight, stunting, and wasting) of children were estimated at 6 years of age using predicted data by the best-fitting model. Children with weight-for-age-z-score <-2 were classified as underweight, those with height-for-age-z-score <-2 as stunted, and those with weight-for-height-z-score <-2 were classified as wasted[46].

Comparison of Goodness of Fit

The three non-nested models were compared using the Akaike information criterion (AIC) and Bayesian information criterion (BIC). Lower AIC and BIC values indicate a better fit. Additionally, residuals of the models were estimated by subtracting observed measurements (weight and height) from the model-predicted ones. The distribution of the residuals of the

For peer review only

models was plotted against age. The residual standard deviation for each model was also reported.

RESULTS

We fitted the three models to weight growth data of 961 children from birth to six years of age separately for boys and girls (Table 1). Children had between 2 and 11 weight and height measurements (Table 1), and 75% of girls had at least four measurements (five in boys).

Table 1 Number of anthropometric measures per child and per sex

Gender	n	Growth dimension	Measures					
			N	Min	Q1	Median	Q3	Max
Girls	500	Weight	2680	2	4	6	9	11
	500	Length/Height	2680	2	4	6	9	11
Boys	461	Weight	2514	2	5	6	9	11
	461	Length/Height	2514	2	5	6	9	11

N: total number of measurements; Min: minimum; Q1: first quartile; Q3: 3rd quartile, Max: maximum.

The distribution of the residuals over age for weight and height of both boys and girls showed that, overall, residuals were centered around zero for all ages and there was no strong dependence of residual distribution over ages for all the models (Figure 1 and figure).

Among the three models, the Jenss-Bayley model presented the closest better fit to the weight and height data, both for girls and boys as illustrated by the lowest AIC and BIC values (Table 2). For both weight and height models, residual standard deviations (RSD) were lowest for the Jenss-Bayley model (Table 2).

Table 2 Comparison of the goodness of fit of the three candidate models

For peer review only

Model	Boys				Girls			
	RSD	AIC	BIC	Log-likelihood	RSD	AIC	BIC	Log-likelihood
Weight								
Jenss	0.46	5251	5300	-2613	0.46	5452	5503	-2714
Reed	0.52	5387	5424	-2684	0.51	5581	5619	-2781
Adapted Gompertz	0.49	5368	5405	-2675	0.49	5587	5625	-2784
Length/Height								
Jenss	1.90	11786	11823	-5884	2.00	12711	12749	-6347
Reed	2.00	11794	11831	-5888	2.10	12739	12777	-6360
Adapted Gompertz	1.95	11792	11829	-5887	2.10	12743	12781	-6362

RSD = Residual standard deviation, AIC = Akaike information criterion, BIC= Bayesian information criterion

Using the Jenss-Bayley model weight and height were estimated for all children at age points where it was not measured, as well as instantaneous growth velocities at different age points, and growth trajectories were calculated (Table 3, 4 and figure 3 respectively).

Table 3 Predicted weight velocities (SD) of girls and boys aged 0 to 6 years, from the Jenss-Bayley model

Age	Boys (n = 461)		Girls (n = 500)	
	Predicted Weight, kg	Velocity, kg/month	Predicted Weight, kg	Velocity, kg/month
3 mo	5.64 (0.62)	0.64 (0.09)	5.18 (0.58)	0.57 (0.58)
6 mo	7.13 (0.76)	0.40 (0.05)	6.58 (0.72)	0. 38 (0.05)
12 mo	8.78 (0.83)	0.30 (0.03)	8.25 (0.77)	0.21 (0.03)
2 yr	10.63 (0.91)	0.14 (0.02)	10. 22 (0.86)	0.12 (0.02)
4 yr	13.65 (1.21)	0.12 (0.02)	13.25 (1.23)	0.12 (0.02)
6 yr	16.63 (1.62)	0.12 (0.02)	16.16 (1.73)	0.12 (0.02)

Table 4 Predicted length/height velocities (SD) of girls and boys aged 0 to 6 years, from the Jenss-Bayley model

Boys (n = 461)	Girls (n = 500)

Age	Predicted Height, cm	Velocity, cm/month	Predicted, cm	Velocity, cm/month
3 mo	59.92 (1.40)	2.67 (0.10)	48.63 (2.29)	2.55 (0.16)
6 mo	66.32 (2.76)	1.66 (0.06)	64.81 (2.98)	1.66 (0.08)
12 mo	73.42 (2.93)	0.91 (0.06)	72.07 (1.87)	0.92 (0.06)
2 yr	82.63 (3.26)	0.63 (0.05)	80.85 (2.13)	0.65 (0.04)
4 yr	96.63 (3.03)	0.61 (0.05)	95.85 (2.87)	0.62 (0.04)
6 yr	111.24 (4.12)	0.61 (0.05)	110.71 (3.72)	0.62 (0.04)

Means and standard deviations (SD) of parameters estimates of the Jenss-Bayley model along with two other candidate models (the adapted Gompertz model and the Reed model) fitted to weight and height of boys and girls are reported in Supplementary Table 1. For the Jenss-Bayley and the adapted Gompertz models, all the parameters highly contributed to describing the mean trajectory since mean parameters were well above their standard deviations.

Nutritional status for all children was estimated at six years. About 1/3 of the children were wasted and underweight (Supplementary Table 2). While 8% of boys and 4% of girls were stunted (Supplementary Table 2). In comparison, in the WHO growth reference population, the percentage of children under -2 Z-scores is expected to be 2.3%

DISCUSSION

We fitted and compared the performance of three growth models (the Jenss, the Reed, and an adaptation of the Gompertz model) to the weight and height of children from birth to around six years of age. There was no strong trend in the residual distribution of the models over age suggesting that all the models performed well without systematic underestimation or overestimation of growth at any age. Comparison of AIC/BIC values and RSD supported the conclusion that the Jenss-Bayley model had the best fit. We, therefore, chose the Jenss-Bayley model to estimate mean growth trajectories and instantaneous growth velocities of children and estimated their nutritional status.

This paper extends the previous limited studies that either fitted the Jenss-Bayley model or compared its goodness of fit with other growth models in childhood. Pagazey and Hauspie[7]

For peer review only

successfully fitted the Jenss-Bayley model on Congolese babies weight data. However, the phase of growth studied in this study was restricted around birth to two years of age. Similarly, Berkey[6] reported that the Jenss-Bayley model fitted well as compared to the Count model on the length and weight data during the early childhood of American children. There were also few studies that reported Reed model fitted well. Simondon and colleagues[8] compared several models on the growth data of 95 Congolese children and reported that the Reed model had a better fit but this study did not include the Jenss-Bayley and the adapted Gompertz models and studied child growth only during infancy. Chirwa and colleagues[28] reported that the Reed model fitted well on the growth data from birth to 10 years in 453 children living in an urban setting in South Africa. The study reported that the Jenss-Bayley model did not fit well in the early years. However, this study included children from an urban setting and did not test the performance of the Gompertz model. In summary, the above previous studies from SSA[6-8, 28] either tested growth models during a limited phase of growth in early childhood or studied children only from a urban setting. The current study differs from the aforementioned studies in a number of respects. First, it included growth data of both infancy and early childhood. Second, it included children from a semi-rural setting which have not been studied before very well in SSA, while the growth and nutrition status of children living in urban setting could differ from those living in rural settings[5, 47]. Third, the growth data used in this study were taken from a prospective study, therefore, it is likely to be more accurate than data from routine surveys or health records. A limitation of this study is that we had lesser measurements between 3 and 5 years of age but as during this period weight and height are known to increase linearly with age, the impact on the obtained growth modelling parameters is likely to be low.

Certain factors could influence the models' goodness of fit when comparing several models. For example, a temporary delay to reach the maximum growth velocity after birth might cause difficulty in modelling weight by models like the Jenss-Bayley model that do not

For peer review only

consider this pattern. We tested the adapted Gompertz model as a potential way to tackle this issue but our adaptation did not perform better than the Jenss-Bayley model. On the other hand, reduced measurement errors as a result of better quality data collection may decrease residuals and hence has an impact on the models' goodness of fit[8].

The estimated instantaneous growth velocities reflected the overall expected pattern of growth velocity in children i.e. a maximum during the earliest age then a decrease until two years and a constant velocity afterward as also reported in other population of children[48, 49], although growth rate could differ depending on the population e.g. weight velocity among boys at 3 years (mean +/- SD): 0.19 ± 0.06 kg/month in American children[48] vs. 0.16 ± 0.04 kg/month in French children respectively[49] and weight velocity among boys at 4 years: 0.17 ± 0.02 kg/month in South African children[50]. However, the study by Regnault et al. excluded birth weight from their analyses while we included the birth weight which could have underestimated weight growth velocities during infancy in our population[39].

CONCLUSION

This paper demonstrated that the Jenss-Bayley model presented the best fit amongst the three candidate models applied to longitudinal weight and height data of Beninese children between birth and six years. We then used this model to estimate growth trajectories and growth velocities even for children with few measurements. Finally, we estimated that about 1/3 of the children were wasted and underweight at six years. Results of this model will be used to study the determinants of growth trajectories.

Acknowledgements The authors gratefully acknowledge families enrolled in the above mentioned studies and the contributors of the research team and staff.

Contributors SA, JB, and FBL collaborated in the conduct, analysis, interpretation of data, and writing. SA performed the growth modeling under the supervision of JB. FBL, RZ, AG, DC,

For peer review only

MC, AM, JA, NF contributed towards data collection and acquisition; follow up of studies; revision of the paper; and provided technical support. FBL and JB collaborated in the conception, planning, methodology, overall supervision and critical revision of the work. All authors read and approved the final manuscript.

Funding The MiPPAD study was co-funded by the European and Developing Countries Clinical Trials Partnership (EDCTP; IP.2007.31080.002), the Malaria in Pregnancy Consortium and the following national agencies: Instituto de Salud Carlos III (PI08/0564), Spain; Federal Ministry of Education and Research (BMBF FKZ: da01KA0803), Germany; Institut de Recherche pour le Developpement (IRD), France. The "Anaemia in Pregnancy: Etiologies and Consequences" (APEC) study received funding from a grant from the Bill and Melinda Gates Foundation. The TOLIMMUNPAL project was funded by Agence Nationale de la Recherche (ANR). The EXPLORE study was funded by Fondation de France. Shukrullah Ahmadi received funding from a grant from Fondation de France (reference 00079816).

Patient consent Obtained

Competing interest The authors declare no conflict of interest. The funders had no role in the design of the study; in the collection, analyses, or interpretation of data; in the writing of the manuscript and in the decision to publish the results.

Data sharing statement No additional data are available.

Ethics approval The MiPPAD trial was approved by the institutional review board of the University of Abomey-Calavi, Benin. The APEC study was approved by the Ethics Committee of the Faculty of Medicine of Cotonou, Benin. The TOLIMMUNPAL study was approved by the Comité Consultatif de Déontologie et d'Éthique (CCDE) of the Institut de Recherche pour le Développement (IRD, France) and the Ethics Committee of the Faculté des Sciences de la Santé, Université d'Abomey Calavi, Benin (approval number: 43/11/2010/CE/FSS/UAC). The

For peer review only

EXPLORE study was approved by the Research Ethics Committee of Institute of Applied Biomedical Sciences, Benin (Comité d'Ethique de la Recherche, Institut des Sciences Biomédicales Appliquées, CER-ISBA, Benin) (approval number: 87 du 17/05/2016).

REFERENCES

1. Moya-Alvarez, V., et al., *Elevated Blood Lead Levels Are Associated with Reduced Risk of Malaria in Beninese Infants.* PLoS One, 2016. **11**(2): p. e0149049.
2. Bodeau-Livinec, F., et al., *Elevated Blood Lead Levels in Infants and Mothers in Benin and Potential Sources of Exposure.* Int J Environ Res Public Health, 2016. **13**(3).
3. Accrombessi, M., et al., *Malaria in Pregnancy Is a Predictor of Infant Haemoglobin Concentrations during the First Year of Life in Benin, West Africa.* PLoS One, 2015. **10**(6): p. e0129510.
4. GBD 2016 Risk Factors Collaborators, *Global, regional, and national comparative risk assessment of 84 behavioural, environmental and occupational, and metabolic risks or clusters of risks, 1990–2016: a systematic analysis for the Global Burden of Disease Study 2016.* 2017.
5. Fotso, J.C., *Child health inequities in developing countries: differences across urban and rural areas.* Int J Equity Health, 2006. **5**: p. 9.
6. Berkey, C.S., *Comparison of two longitudinal growth models for preschool children.* Biometrics, 1982. **38**(1): p. 221-34.
7. Pagezy, H. and R. Hauspie, *Growth in weight of African babies, aged 0–24 months, living in a rural area at the Lake Tumba, Zaire.* Annals of Tropical Paediatrics, 1985. **5**(1): p. 41-47.
8. Simondon, K.B., et al., *Comparative study of five growth models applied to weight data from congolese infants between birth and 13 months of age.* American Journal of Human Biology, 1992. **4**(3): p. 327-335.
9. Deming, J., *Application of the Gompertz curve to the observed pattern of growth in length of 48 individual boys and girls during the adolescent cycle of growth.* Hum Biol, 1957. **29**(1): p. 83-122.
10. Laird, A.K., *Evolution of the human growth curve.* Growth, 1967. **31**(4): p. 345-55.
11. Adair, L.S., et al., *Size at birth, weight gain in infancy and childhood, and adult blood pressure in 5 low- and middle-income-country cohorts: when does weight gain matter?* Am J Clin Nutr, 2009. **89**(5): p. 1383-92.
12. Cameron, N., et al., *Timing and magnitude of adolescent growth in height and weight in Cape coloured children after kwashiorkor.* J Pediatr, 1986. **109**(3): p. 548-55.
13. Fetuga, M.B., et al., *Growth pattern of schoolchildren in Sagamu, Nigeria using the CDC standards and 2007 WHO standards.* Indian Pediatr, 2011. **48**(7): p. 523-8.
14. Guedes, D.P., et al., *Physical growth of schoolchildren from the Jequitinhonha Valley, Minas Gerais, Brazil: Comparison with the CDC-2000 reference using the LMS method.* Ann Hum Biol, 2010. **37**(4): p. 574-84.
15. Hauspie, R.C. and H. Pagezy, *Longitudinal Study of Growth of African Babies: An Analysis of Seasonal Variations in the Average Growth Rate and the Effects of Infectious Diseases on Individual and Average Growth Patterns.* Acta Paediatrica, 1989. **78**(s350): p. 37-43.
16. Johnson, W., et al., *Using the WHO 2006 child growth standard to assess the growth and nutritional status of rural south Indian infants.* Ann Hum Biol, 2012. **39**(2): p. 91-101.
17. Kalanda, B.F., et al., *Catch-up growth in Malawian babies, a longitudinal study of normal and low birthweight babies born in a malarious endemic area.* Early Hum Dev, 2005. **81**(10): p. 841-50.

For peer review only

18. Maleta, K., et al., *Seasonality of growth and the relationship between weight and height gain in children under three years of age in rural Malawi.* Acta Paediatrica, 2007. **92**(4): p. 491-497.
19. Mushtaq, M.U., et al., *Height, weight and BMI percentiles and nutritional status relative to the international growth references among Pakistani school-aged children.* BMC Pediatrics, 2012. **12**(1).
20. Olusanya, B.O. and J.K. Renner, *Predictors of growth velocity in early infancy in a resource-poor setting.* Early Hum Dev, 2011. **87**(10): p. 647-52.
21. Simondon, K.B., et al., *Comparative study of five growth models applied to weight data from congolese infants between birth and 13 months of age.* Am J Hum Biol, 1992. **4**(3): p. 327-335.
22. Stein, A.D., et al., *Growth patterns in early childhood and final attained stature: data from five birth cohorts from low- and middle-income countries.* Am J Hum Biol, 2010. **22**(3): p. 353-9.
23. Adair, L.S., et al., *Associations of linear growth and relative weight gain during early life with adult health and human capital in countries of low and middle income: findings from five birth cohort studies.* Lancet, 2013. **382**(9891): p. 525-34.
24. Khan, A.I., et al., *Effects of exclusive breastfeeding intervention on child growth and body composition: the MINIMat trial, Bangladesh.* Acta Paediatr, 2013. **102**(8): p. 815-23.
25. Khan, A.I., et al., *Early invitation to food and/or multiple micronutrient supplementation in pregnancy does not affect body composition in offspring at 54 months: follow-up of the MINIMat randomised trial, Bangladesh.* Matern Child Nutr, 2015. **11**(3): p. 385-97.
26. Stevens, G.A., et al., *Trends in mild, moderate, and severe stunting and underweight, and progress towards MDG 1 in 141 developing countries: a systematic analysis of population representative data.* Lancet, 2012. **380**(9844): p. 824-34.
27. Mramba, L., et al., *A growth reference for mid upper arm circumference for age among school age children and adolescents, and validation for mortality: growth curve construction and longitudinal cohort study.* BMJ, 2017. **358**: p. j3423.
28. Chirwa, E.D., et al., *Multi-level modelling of longitudinal child growth data from the Birth-to-Twenty Cohort: a comparison of growth models.* Ann Hum Biol, 2014. **41**(2): p. 168-79.
29. Rozzi, F.V., et al., *Growth pattern from birth to adulthood in African pygmies of known age.* Nat Commun, 2015. **6**: p. 7672.
30. Padonou, G., et al., *Factors associated with growth patterns from birth to 18 months in a Beninese cohort of children.* Acta Trop, 2014. **135**: p. 1-9.
31. Gonzalez, R., et al., *Intermittent preventive treatment of malaria in pregnancy with mefloquine in HIV-negative women: a multicentre randomized controlled trial.* PLoS Med, 2014. **11**(9): p. e1001733.
32. Ouedraogo, S., et al., *Maternal anemia at first antenatal visit: prevalence and risk factors in a malaria-endemic area in Benin.* Am J Trop Med Hyg, 2012. **87**(3): p. 418-24.
33. d'Almeida, T.C., et al., *Soluble human leukocyte antigen -G during pregnancy and infancy in Benin: Mother/child resemblance and association with the risk of malaria infection and low birth weight.* PLoS One, 2017. **12**(2): p. e0171117.
34. Ahmadi, S., et al., *Hunting, Sale, and Consumption of Bushmeat Killed by Lead-Based Ammunition in Benin.* Int J Environ Res Public Health, 2018. **15**(6).
35. Bodeau-Livinec, F., et al., *Neurocognitive testing in West African children 3-6 years of age: Challenges and implications for data analyses.* Brain Research Bulletin, 2018.
36. World Health Organization, *Physical Status: the use and interpretation of anthropometry. A report of a WHO Expert Committee.* , in *WHO Technical Series, Geneva.* 1995, WHO.
37. World Health Organization, *The WHO Child Growth Standards*. 2006 [cited 2019.
38. Jenss, R.M. and N. Bayley, *A mathematical method for studying the growth of a child.* Human Biology, 1937. **9**(4): p. 556.

For peer review only

39. Botton, J., et al., *Postnatal weight and height growth modeling and prediction of body mass index as a function of time for the study of growth determinants.* Ann Nutr Metab, 2014. **65**(2-3): p. 156-66.
40. Karkach, A., *Trajectories and models of individual growth.* Demographic Research, 2006. **15**: p. 347-400.
41. Tjørve, K.M.C. and E. Tjørve, *The use of Gompertz models in growth analyses, and new Gompertz-model approach: An addition to the Unified-Richards family.* PLOS ONE, 2017. **12**(6): p. e0178691.
42. Winsor, C.P., *The Gompertz Curve as a Growth Curve.* Proc Natl Acad Sci U S A, 1932. **18**(1): p. 1-8.
43. Berkey, C.S. and R.B. Reed, *A model for describing normal and abnormal growth in early childhood.* Hum Biol, 1987. **59**(6): p. 973-87.
44. Hauspie, R.C., *Mathematical models for the study of individual growth patterns.* Rev Epidemiol Sante Publique, 1989. **37**(5-6): p. 461-76.
45. Comets, E., A. Lavenu, and M. Lavielle, *Parameter Estimation in Nonlinear Mixed Effect Models Using saemix, an R Implementation of the SAEM Algorithm.* Journal of Statistical Software, 2017. **80**(3): p. 1-42.
46. Organisation, W.H. *The WHO Child Growth Standards*. 2006 [cited 2019 2019 02 February]; Available from: https://www.who.int/childgrowth/standards/en/.
47. Kennedy, G., et al., *Does living in an urban environment confer advantages for childhood nutritional status? Analysis of disparities in nutritional status by wealth and residence in Angola, Central African Republic and Senegal.* Public Health Nutrition, 2007. **9**(2): p. 187-193.
48. Regnault, N., et al., *Comparative Study of Four Growth Models Applied to Weight and Height Growth Data in a Cohort of US Children from Birth to 9 Years.* Annals of Nutrition and Metabolism, 2014. **65**(2-3): p. 167-174.
49. Botton, J., et al., *Postnatal weight and height growth velocities at different ages between birth and 5 y and body composition in adolescent boys and girls.* Am J Clin Nutr, 2008. **87**(6): p. 1760-8.
50. Chirwa, E.D., et al., *Postnatal growth velocity and overweight in early adolescents: a comparison of rural and urban African boys and girls.* Am J Hum Biol, 2014. **26**(5): p. 643-51.

FIGURE LEGENDS

Figure 1 Residuals of weight models for boys and girls

(Data of the first two deciles was gathered, as there were more than 10% of the data at birth)

Figure 2 Residuals of height models for boys and girls

(Data of the first two deciles was gathered, as there were more than 10% of the data at birth)

Figure 3 Weight and height mean growth trajectories from the Jenss-Bayley model. (A) Weight growth curve. (B) Height growth curve. The solid (black) line implies boys curve and dashed line implies girls

For peer review only

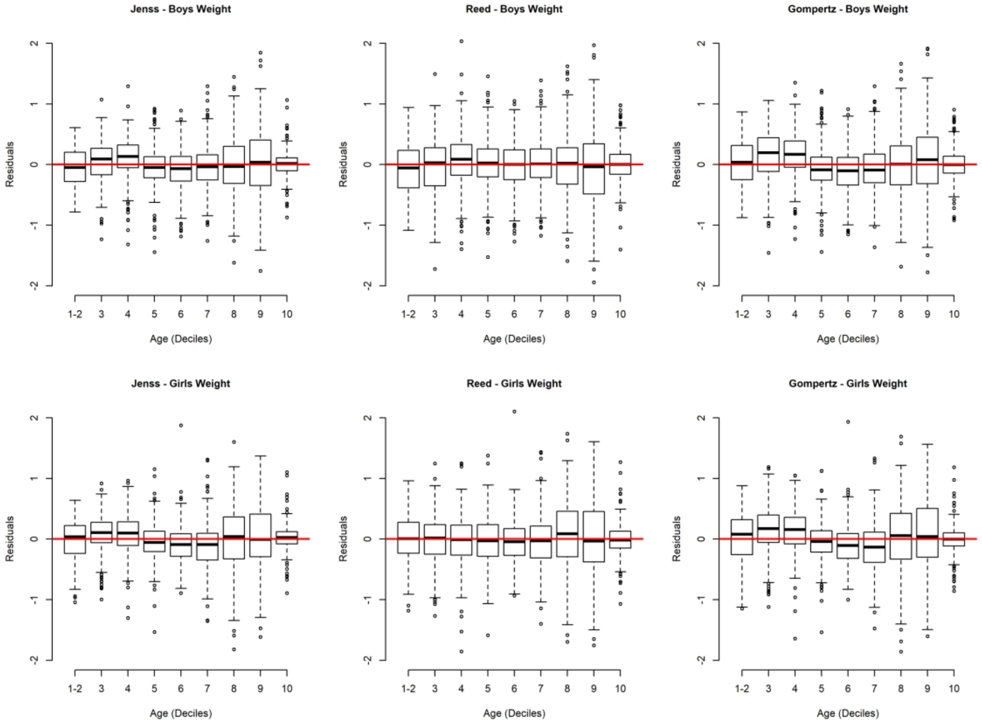

Figure 1 Residuals of weight models for boys and girls
(Data of the first two deciles was gathered, as there were more than 10% of the data at birth)

270x202mm (300 x 300 DPI)

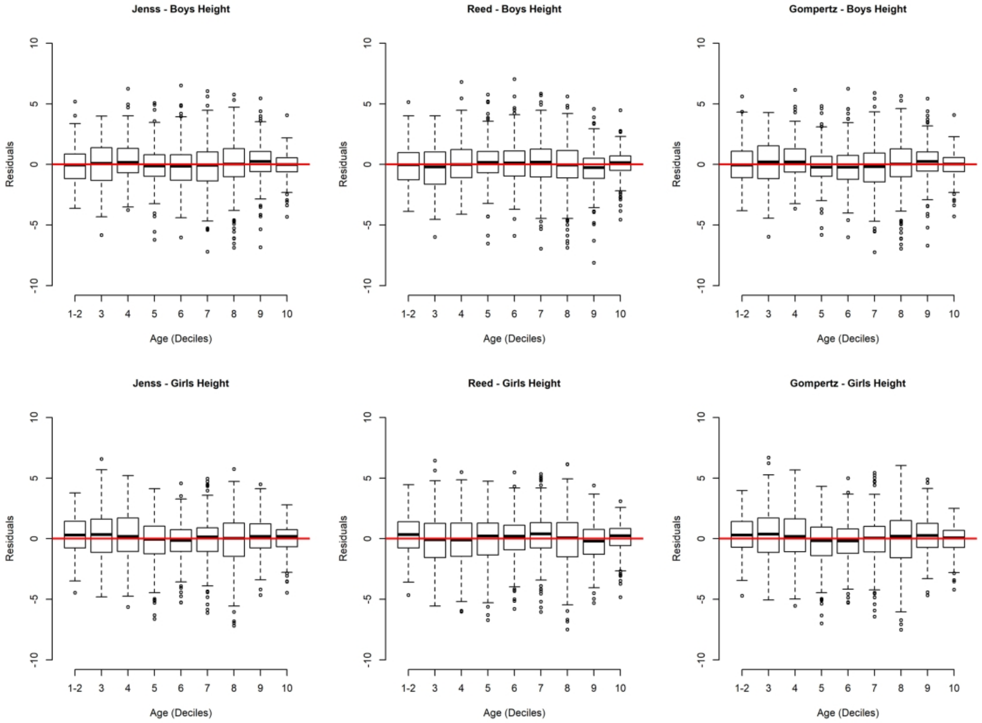

Figure 2 Residuals of height models for boys and girls
(Data of the first two deciles was gathered, as there were more than 10% of the data at birth)

270x202mm (300 x 300 DPI)

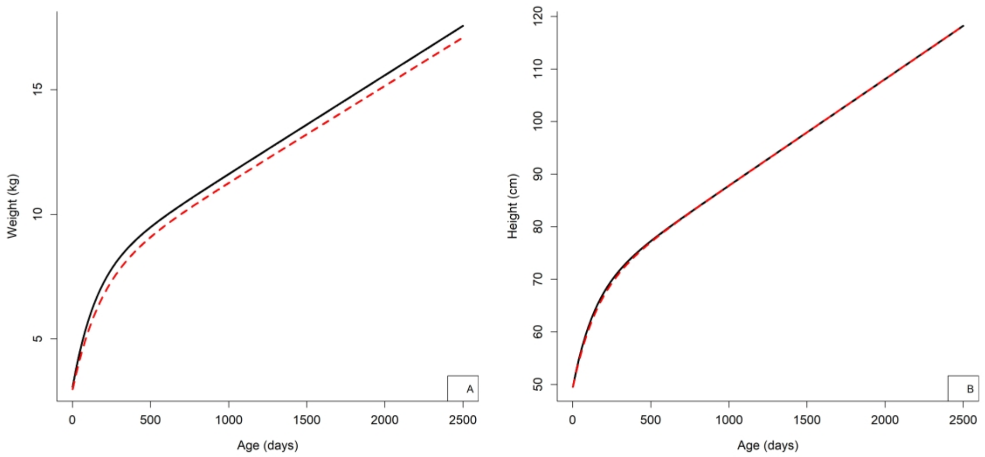

Figure 3 Weight and height mean growth trajectories from the Jenss-Bayley model. (A) Weight growth curve. (B) Height growth curve. The solid (black) line implies boys curve and dashed line implies girls

399x202mm (300 x 300 DPI)

Supplementary tables

Supplementary Table 1 Parameters estimates for three candidate models [a] fitted to weight and height

Model	Parameters	Boys		Girls	
		Mean	SD	Mean	SD
Weight models					
Jenss					
	A	1.11	0.05	1.08	0.06
	B	5.53	0.15	5.55	0.18
	C	1.52	0.16	1.49	0.15
	D	4.97	0.24	5.15	0.37
Adapted Gompertz	A	2.02	0.08	1.96	0.07
	B	0.90	<0.01	0.85	< 0.01
	C	4.67	0.24	4.85	0.25
	D	5.50	0.13	5.49	0.15
Reed	A	-1.18	<0.01	-0.89	0.12
	B	-5.87	0.18	-5.78	0.18
	C	0.39	0.08	0.28	0.07
	D	1.44	<0.01	1.34	<0.01
Height models					
Jenss					
	A	3.90	0.02	3.90	0.02
	B	3.90	0.08	3.90	0.06
	C	2.90	0.01	2.90	0.06
	D	4.90	0.09	5.00	0.07
Adapted Gompertz	A	4.21	0.02	4.2	0.02
	B	0.31	< 0.01	0.3	<0.01
	C	4.83	0.13	4.9	0.12
	D	3.92	0.07	3.9	0.06
Reed	A	3.50	0.03	3.5	0.03
	B	-4.20	0.09	-4.2	0.08
	C	1.80	0.02	1.7	0.02
	D	2.80	< 0.01	2.8	<0.01

For peer review only

For peer review only - http://bmjopen.bmj.com/site/about/guidelines.xhtml

[a] the sign of the parameters for the adapted Gompertz and the Reed model was constrained by using exponential functions, as shown in equation 2 for the Jenss-Bayley model.

Supplementary Table 2 Stunting, Underweight, Wasting in children using predicted data from the Jenss-Bayley model. n (%)

Sex	Underweight (WAZ<–2)	Stunting (HAZ<-2)	Wasting (WHZ<-2)
Boys (n=461)	161 (35)	37 (8)	125 (27)
Girls (n=500)	168 (34)	22 (4)	190 (38)

For peer review only

For peer review only - http://bmjopen.bmj.com/site/about/guidelines.xhtml

BMJ Open

Comparison of Growth Models to Describe Growth from Birth to 6 Years in a Beninese Cohort of Children with Repeated Measurements

Journal:	*BMJ Open*
Manuscript ID	bmjopen-2019-035785.R1
Article Type:	Original research
Date Submitted by the Author:	07-Apr-2020
Complete List of Authors:	AHMADI, Shukrullah; INSERM, INRA, Université de Paris, Centre of Research in Epidemiology and Statistics /CRESS<br>Bodeau-Livinec, Florence; EHESP; INSERM, INRA, Université de Paris, Centre of Research in Epidemiology and Statistics /CRESS<br>Zoumenou, Roméo ; Institut de Recherche pour le Développement (IRD), Université Paris Descartes, MERIT (Mère et Enfant Face aux Infections Tropicales)-UMR 216<br>Garcia, Andre; Institut de Recherche pour le Développement (IRD), Université Paris Descartes, MERIT (Mère et Enfant Face aux Infections Tropicales)-UMR 216<br>Courtin, David; Institut de Recherche pour le Développement (IRD), Université Paris Descartes, MERIT (Mère et Enfant Face aux Infections Tropicales)-UMR 216<br>Alao, Jules; CHU-MEL Hospital<br>Fievet, Nadine ; Institut de Recherche pour le Développement (IRD), Université Paris Descartes, MERIT (Mère et Enfant Face aux Infections Tropicales)-UMR 216<br>Cot, Michel; Institut de Recherche pour le Développement (IRD), Université Paris Descartes, MERIT (Mère et Enfant Face aux Infections Tropicales)-UMR 216<br>Massougbodji, Achille; Université d'Abomey-Calavi Faculté des Sciences de la Santé<br>Botton, Jérémie ; EPI-PHARE, Epidemiology of health products
**Primary Subject Heading**:	Epidemiology
Secondary Subject Heading:	Paediatrics, Public health, Nutrition and metabolism, Global health
Keywords:	STATISTICS & RESEARCH METHODS, EPIDEMIOLOGY, PAEDIATRICS, PUBLIC HEALTH

SCHOLARONE™
Manuscripts

I, the Submitting Author has the right to grant and does grant on behalf of all authors of the Work (as defined in the below author licence), an exclusive licence and/or a non-exclusive licence for contributions from authors who are: i) UK Crown employees; ii) where BMJ has agreed a CC-BY licence shall apply, and/or iii) in accordance with the terms applicable for US Federal Government officers or employees acting as part of their official duties; on a worldwide, perpetual, irrevocable, royalty-free basis to BMJ Publishing Group Ltd ("BMJ") its licensees and where the relevant Journal is co-owned by BMJ to the co-owners of the Journal, to publish the Work in this journal and any other BMJ products and to exploit all rights, as set out in our licence.

The Submitting Author accepts and understands that any supply made under these terms is made by BMJ to the Submitting Author unless you are acting as an employee on behalf of your employer or a postgraduate student of an affiliated institution which is paying any applicable article publishing charge ("APC") for Open Access articles. Where the Submitting Author wishes to make the Work available on an Open Access basis (and intends to pay the relevant APC), the terms of reuse of such Open Access shall be governed by a Creative Commons licence – details of these licences and which Creative Commons licence will apply to this Work are set out in our licence referred to above.

Other than as permitted in any relevant BMJ Author's Self Archiving Policies, I confirm this Work has not been accepted for publication elsewhere, is not being considered for publication elsewhere and does not duplicate material already published. I confirm all authors consent to publication of this Work and authorise the granting of this licence.

For peer review only

Comparison of Growth Models to Describe Growth from Birth to 6 Years in a Beninese Cohort of Children with Repeated Measurements

Ahmadi S [1] , Bodeau-Livinec F [1,2] , Zoumenou R[3], Garcia A[3], Courtin D[3], Jules A[4], Fievet N[3], Cot M[3,], Massougbodji A[5], Botton J[6].

[1] Université de Paris, Centre of Research in Epidemiology and Statistics /CRESS, INSERM, INRA, F-75004, Paris, France. shukrullah.ahmadi@inserm.fr.
[2] EHESP, F-35000 Rennes, France. florence.bodeau-livinec@ehesp.fr
[3] MERIT (Mère et Enfant Face aux Infections Tropicales)-UMR 216, Institut de Recherche pour le Développement (IRD), Université Paris Descartes, 75006 Paris, France. zoumenour@yahoo.fr; andre.garcia@ird.fr; david.courtin@ird.fr; nadine.fievet@ird.fr; michel.cot@ird.fr
[4] Paediatric Department, Mother and Child University and Hospital Center (CHU-MEL), Cotonou, Benin. amomj@yahoo.fr
[5] Faculté des Sciences de la Santé, Université d'Abomey-Calavi, Cotonou, Benin. massougbodjiachille@yahoo.fr
[6] EPI-PHARE, Epidemiology of health products, Saint-Denis, France. jeremie.botton@ansm.sante.fr

Correspondence to Shukrullah Ahmadi, Centre of Research in Epidemiology and Statistics /CRESS. INSERM, INRA, F-75004, Paris, France. shukrullah.ahmadi@inserm.fr

Word count: ≈ 4080

For peer review only

ABSTRACT

Objective To select a growth model that best describes individual growth trajectories of children and to present some growth characteristics of this population.

Settings Participants were selected from a prospective cohort conducted in three health centres (Allada, Sekou and Attogon) in a semi-rural region of Benin, Sub-saharan, Africa.

Participants Children from birth to six years of age recruited in a cohort study with at least two valid height and weight measurements were included (n=961).

Primary and secondary outcome measures This study compared the goodness of fit of three structural growth models (Jenss-Bayley, Berkey-Reed and a newly adapted version of the Gompertz growth model) on longitudinal weight and height growth data of boys and girls. The goodness-of-fit of the models was assessed using residual distribution over age and compared with the Akaike Information Criterion (AIC) and Bayesian Information Criterion (BIC). The best-fitting model allowed estimating mean weight and height growth trajectories, individual growth and growth velocities. Underweight, stunting and wasting were also estimated at 6 years.

Results The three models were able to fit well both weight and height data. The Jenss-Bayley model presented the best fit for weight and for height, both in boys and girls. Mean height growth trajectories were identical in shape and direction for boys and girls while mean weight growth curve of girls fell slightly below the curve of boys after neonatal life. Finally, 35%, 27.7% and 8% of boys; and 34%, 38.4%, and 4% of girls were estimated to be underweight, wasted, and stunted at 6 years of age, respectively.

Conclusion The growth parameters of the best fitting Jenss-Bayley model can be used to describe growth trajectories and study their determinants

For peer review only

Keywords Weight, height, growth modelling, mixed-effects modelling, structural growth models, sub-Saharan Africa, Growth trajectory

Strengths and limitations of this study

- This is the first study to assess the comparative merits of growth models on child anthropometric data from birth to six years of age in a semi-rural setting in sub-Saharan Africa with multiple repeated measures in a population with a high prevalence of undernutrition.
- A new model which is an adaptation of the Gompertz model has been described and evaluated to test whether it shows a better fit compared to some known growth models previously used.
- This modelling technique allowed predicting weight and height for all children at several age points even when not measured; and its usefulness is showed in calculating mean and individual growth trajectories and velocities, and in assessing the extent of undernutrition (underweight, stunting and wasting).
- The generalizability of these results cannot be guaranteed because the study sample is not representative of the Beninese children.
- Other models could have been tested but the overall fit of the selected models was very optimal and they present advantages for future studies in this population.

For peer review only

INTRODUCTION

Insufficient growth during childhood adversely affects later health outcomes. According to estimates from the Global Burden of Diseases, Injuries, and Risk Factors Study 2016[1], the prevalence of stunting, wasting, and underweight (based on the definitions using the WHO 2006 growth standards) in children under five were, respectively, 36.6%, 8.6% and 19.5% in sub-Saharan Africa (SSA) in 2015. Furthermore, more than 23% of under-five mortality was attributable to child growth failure, being thus the second leading risk factor for child mortality in this region.

Mathematical growth modelling is a powerful tool for the study of child growth and growth trajectories. It consists of fitting models to physical growth data (e.g. weight, length/height, head circumference) to obtain an appropriate growth curve that will conveniently summarize growth information provided by weight and height measurements of children, even from irregularly spaced growth measurements[2]. Clinicians routinely compare growth data to growth charts to identify impaired growth trajectories in children. Indeed, looking at growth trajectories (weight, height/length or BMI) has gained importance for several purposes. It is becoming essential for surveillance to identify abnormal growth trajectories[3]. In the post-natal period, length/height and weight surveillance is an essential tool for monitoring child growth. It is also used in research to study determinants of growth including identification of modifiable risk factors and sensitive age periods during which interventions may be especially useful to achieve optimal growth[4]. Characterizing growth trajectories is also important to study whether past growth is associated with future growth patterns or later health outcomes[5].

There are several structural and non-structural growth models to describe child growth, amongst them the Jenss-Bayley model[6], the Reed model[7], and the Gompertz functions[8, 9]. These three models are amongst the most common structural models

For peer review only

described in the literature, while non-structural models are mainly polynomial and splines[10-13], as cited in Chirwa et al. (2014)[14]. The parameters of structural models have a biological basis. In contrast, non-structural models do not formulate any particular form of growth curve and may demonstrate instability at extremities and. Besides, the parameters obtained from non-structural models do not provide any biological interpretation[12, 15].

Many studies have analysed child growth data in Africa, but few studies[6, 7, 13, 14, 16-18] have applied quadratic, structural or non-structural growth models on African longitudinal growth data. A summary of some of these studies is presented in Table 1. However, there are limited studies[7, 14] in the literature that have assessed the comparative merits of models like the Jenss-Bayley on child growth in SSA and Gompertz model on child growth in SSA. Furthermore, as to our knowledge, no study has compared the fitness of the adapted Gompertz growth model on child growth data.

This study aimed to compare the fit of three structural growth models (the Jenss-Bayley model, the Reed model, and a new model which is an adaptation of the Gompertz model) applied to weight and height growth data in a population of Beninese children between birth and 6 years of age, living in a semi-rural region and to describe their growth using the selected model.

Table 1. Summary of some studies that have used quadratic, structural and non-structural models to describe physical growth

Reference	Study population	Model(s) used	Period of growth
Pagazey and Hauspie (1985)	Congolese	Jenss-Bayley	0 – 24 months
Simondon et al. (1992)	Congolese	Berkey-Reed 1st order; Count; Karlberg; Berkey-Reed 2nd order; Kouchi	0 – 13 months

For peer review only

Table 1 continued

Cameron et al. (1986)	South-African	Preece-Baines	5 months – 4.5 years (cases); 15-year follow-up (controls)
Olusanya and Renner (2011)	Nigerian	2-point Birthweigth Model; 2-point Average Weight Model; Exponential Model	Early infancy
Chirwa et al. (2014)	South-African	Berkey-Reed; Count; Jenss-Bayley; the adapted Jenss-Bayley; 2nd and 3rd order Polynomial models	birth - early childhood
Padonou et al. (2014)	Beninese	Quadratic	0 – 18 months
Rozzi et al. (2015)	Baka Pygmies	Jenss–Bayley model; Reed; Count–Gompertz model; the Preece-Baines models	0 – 25 years

METHODOLOGY

Study design and data sources

Weight and height measurements were available from a prospective cohort study of Beninese children followed from birth to six years of age. These children who participated were born within the MiPPAD clinical trial (Malaria in Pregnancy Preventive Alternative Drugs) and followed in three ancillary studies, the APEC, TOLIMMUNPAL and TOVI studies, and finally assessed in the EXPLORE study at mean six years of age. The research objectives, design, sample size, and follow-up period varied for these studies.

Participating children were born of HIV-negative mothers (n = 1182) enrolled in the MiPPAD clinical trial (Malaria in Pregnancy Preventive Alternative Drugs) (NCT00811421)

For peer review only

in 2011 in the district of Allada, south Benin[19]. The MiPPAD trial, which enrolled pregnant women before 28 weeks of gestation at the first antenatal care visit, compared the efficacy of two intermittent preventive treatments for malaria in pregnancy (IPTp). Height and weight of children were assessed at birth and women were requested to bring their infants to the health centre when the babies were 1 month, and also at 9 and 12 months of age. Children included in our analyses met the following inclusion criteria: being born within the MiPPAD clinical trial, and assessed at least twice with valid anthropometric measurements (weight and height) between birth and six years of age within MiPPAD, APEC, TOLIMMUNPAL, TOVI or EXPLORE studies.

Within this (MiPPAD) cohort, a sub-sample of 400 children was followed more closely. These children were followed from birth to two years of age in the APEC (Anemia in pregnancy: etiology and consequences)[20] and TOLIMMUNPAL studies[21]. The APEC study included the first 400 offspring born within the MiPPAD study including anthropometric measurements of children at 6 and 9 months. Infants assessed in the APEC study were also followed-up in the TOLIMMUNPAL study between birth and 24 months with further anthropometric measurements at 15, 18, 21 and 24 months.

The TOVI study followed-up at one year-of-age 747 singleton children born within MiPPAD. Among these children, only 92 children randomly selected were assessed between 3 and 5 years of age for anthropometric measurements[22]. Finally, at 6 years of age, children were assessed within the EXPLORE study at mean six years of age for anthropometric measurements (2016 -2018)[23, 24]. Supplementary Figure 1 shows participants inclusion and how different ancillary studies are embedded in this cohort.

Appropriate participant consent and ethics approval were obtained for the different studies included.

For peer review only

Patient and public involvement

Patients and the public were not directly involved in the planning of this study. Results will be communicated to participants via community meetings with local leaders.

Anthropometric measurement

Measurements were performed by trained staff at each visit, with children wearing light clothes only and no shoes. From birth up to around 2 years, infants' length was measured to the nearest 1 mm using a locally manufactured wooden measuring scale according to the WHO guidelines[25]. From birth up to 2 years, infants' weight was measured using an electronic baby scale (SECA type 354) with a precision of 10 g. From 2 years onwards, standing weight and height was measured for all children. Height was measured by a locally manufactured length board. The length board comprised of a measuring board mounted to the wall with an attached measuring tape and moveable right angle headpiece. Weight was measured using an analogue scale (CAMRY DT602). At six years, children were weighted with the Tanita scale.

Data inclusion

Participants with at least two weight or height measurements anytime between birth and around six years of age were included in the analysis. Weight-for-age z-scores (WAZ) and height-for-age z-scores (HAZ) were calculated based on the WHO growth standards[26]. To identify outliers, as the distribution of the Z-scores was shifted to the left, the distribution was corrected it by centring it to the mean. Then measurements with centred WAZ or HAZ greater than +4 or less than -4 were considered outliers for growth and were systematically excluded from the analysis. Additionally, individual weight and height profiles were plotted to visually check individual growth trajectories for plausibility. 65 height (out of 5291 measures) and 35 weight observations (out of 5291 measures) were excluded. They were considered as outliers on the descriptive graph even after the systematic removal of

For peer review only

observations with WAZ or HAZ greater than +4 or less than -4. The final analyses had 961 participants (461 boys and 500 girls). The number of children with anthropometric measurements at different age intervals is presented in the supplementary Table 1.

Growth models

The following three structural growth models were compared: the Jenss-Bayley model, the Reed model, and a new model which is an adaptation of the Gompertz model. These models are presented in the equations 1, 3 and 4, where E (y_{ij}) is the expected value (weight or length of child i at j^{th} occasion, t_{ij} is age in days, A_i, B_i C_i and D_i are the parameters of the functions for the ith child.

The Jenss-Bayley model[27] is presented in equation 1 and as another parameterization in equation 2. Briefly, a or exp^{A} is the predicted value at birth (t=0); b or exp^{-B} is the asymptotic slope or growth rate from about two years onwards; d or exp^{-D} allows for decelerating growth (decreasing exponential function) during infancy. The parameter c or exp^{C} can reflect the degree of catch-up growth. These four parameters or model coefficients can be obtained for each individual-thanks to a mixed-effects modelling approach- and represent the global individual growth trajectory. Parameters (or a combination of them, e.g. the velocity) can then be studied in association with the determinants of growth or other independent variables[28].

$$E(y_{ij}) = a_i + b_i \cdot t_{ij} - (exp^{c_i + d_i \cdot t_{ij}}) \qquad (1)$$

$$E(y_{ij}) = exp^{A_i} + exp^{-B_i} \cdot t_{ij} + exp^{C_i} \cdot (1 - exp^{-exp^{-D_i} \cdot t_{ij}}) \qquad (2)$$

The Gompertz model[29] (equation 3 with Di=0) is a 3-parameter model commonly used to model human growth and development[30, 31]. Parameter A_i represents the upper asymptote (maximum value), C_i represents growth rate and B_i is related to weight at birth (t=0). The Gompertz model theoretically approaches a null asymptote at later ages and to

better represent the positive linear growth between about 3 and 6 years of age, the Gompertz growth function[31, 32] was expanded by adding a 4th parameter D_i on height and weight modelling. This adaptation allows the model to have a non-flat asymptote (slope=velocity different from 0). This permits to fit the linear part of the growth at the later ages in our data (constant linear growth velocity). The model is non-linear in the model parameters.

$$E(y_{ij}) = A_i \cdot exp\,(-B_i \cdot exp\,(-C_i \cdot t_{ij})) + D_i \cdot t_{ij} \qquad (3)$$

In the Reed model[33], parameter B_i is the asymptotic slope or growth rate/velocity of preschool growth, C_i is an important component of rapid early childhood growth[2, 34] and models the exponential growth deceleration, D_i is used to better model early life variations in growth, and A_i is the intercept. The model is as follows:

$$E(y_{ij}) = A_i + B_i.t_{ij} + C_i.ln\,(t_{ij} + 1) + \frac{D_i}{t_{ij}} \qquad (4)$$

Statistical methods

The Jenss-Bayley model, the Reed model, and the adapted Gompertz model were applied to weight and height growth data of children from birth to 6 years of age.

The Reed fixed-effects model was fit using the lm (linear model) function in R[35], and the Jenss and Gompertz models using the nls (Nonlinear Least Squares) function in R [35] to obtain initial values more easily[28]. Then the models were fitted to weight and height data using the SAEMIX[36] package in R-3.5.1 software[35] as applied in another study[28]. Due to convergence issues with unstructured matrices, a diagonal variance-covariance matrix was selected for random effects.

Growth trajectories of the children were estimated using a recently proposed method described elsewhere[28]. Briefly, weight and height growth trajectories and growth velocities (first derivative of the equation) was estimated using the model presenting the best fit (the Jenss-Bayley model). Fixed-effect parameters (A, B, C and D) obtained from the mixed-

For peer review only

effects model using SAEMIX package were used to represent average growth trajectories. The Jenss-Bayley model then allowed predicting individual weight and height by substituting the individual model parameters into their corresponding model equation (equation 2). Similarly, individual growth velocities were calculated by substituting determined individual parameters into the derivative of the Jenss-Bayley model. As a derivative of the Jenss-Bayley model, the following equation can be used to estimate growth velocity over time[28].

$$dy/dt = exp^{-B_i} + exp^{C_i - D_i - exp^{-D_i} \cdot t_{ij}} \qquad (5)$$

Finally, undernutrition (underweight, stunting, and wasting) of children was estimated at 6 years of age using predicted data by the best-fitting model. Children with weight-for-age-z-score <-2 were classified as underweight, those with height-for-age-z-score <-2 as stunted, and those with weight-for-height-z-score <-2 were classified as wasted[37].

For peer review only

Comparison of Goodness of Fit

The three non-nested models were compared using the Akaike information criterion (AIC), delta AIC, and Bayesian information criterion (BIC). Lower AIC and BIC values indicate a better fit. The delta AIC [38] for a candidate model, is the difference between the AIC values of the best and the candidate models. This difference (delta AIC) is then used as follows to determine the amount of support for each candidate model. If the delta AIC is < 2, there is substantial evidence to support the candidate model (i.e., the candidate model is almost as best performing as the best model). If the delta AIC is between 4 and 7, there is considerably less support for the candidate model to be the best model. Finally, if the delta AIC is >10, there is essentially no support for the candidate model to better than the best model. Additionally, residuals of the models were estimated by subtracting observed measurements (weight and height) from the model-predicted ones. The distribution of the

residuals of the models was plotted against age. The residual standard deviation for each model was also reported.

RESULTS

Three models were fitted to weight growth data of 961 children from birth to six years of age separately for boys and girls (Table 2). Children had between 2 and 11 weight and height measurements (Table 2), and 75% of girls had at least four measurements (five in boys). Some descriptive characteristics of the study population are presented in supplementary table 2. The overall mean maternal age at 1 year of the study child was 25.8 years (s.d. 5.6). Almost ¾ of mothers could not read or write at the start of the cohort. Almost 10% of mothers were underweight before their pregnancy. Almost 10% of boys and 14% of girls had a lower birth weight (<2.5 kg) and less than 7 % of children were born preterm. Almost 10% of children had malaria at 1 year of age and almost 74% of boys and 67% of girls had anaemia at 1 year of age.

Table 2 Number of anthropometric measures per child and sex

Gender	n	Growth dimension	Measures					
			N	Min	Q1	Median	Q3	Max
Girls	500	Weight	2680	2	4	6	9	11
	500	Length/Height	2680	2	4	6	9	11
Boys	461	Weight	2514	2	5	6	9	11
	461	Length/Height	2514	2	5	6	9	11

N: total number of measurements; Min: minimum; Q1: first quartile; Q3: 3rd quartile, Max: maximum.

For peer review only

The distribution of the residuals over age for weight and height of both boys and girls showed that, overall, residuals were centred around zero for all ages and there was no strong dependence of residual distribution over ages for all the models (Figure 1 and Figure 2).

For weight models, the Jenss-Bayley model had the lowest AIC and BIC values, both in boys and girls, indicating a better fit than the two other candidate models (Table 3). Both the Reed and the adapted Gompertz model had high delta AIC values for both boys (136 and 117, respectively) and girls (129 and 135, respectively) indicating essentially no support for these two candidate model to be better than the best fitting model (Jenss-Bayley).

For height models, the Jenss-Bayley model and the adapted Gompertz model had the lowest AIC and BIC values for the height model in boys while the Jenss-Bayley model had the lowest AIC values in girls than the Reed and adapted Gompertz model. Concerning delta AIC values, both the Reed and the adapted Gompertz model had values less than 10 for boys (8 and 6, respectively) indicating considerably less support for these two models to be the best fitting model. While in girls, these two candidate models had delta AIC values >10 (28 and 32, respectively) which does not indicate any support for these two models to have a better fit than the Jenss-Bayley model.

For peer review only

Table 3 Comparison of the goodness of fit of the three candidate models

	Boys					Girls				
**Model**	**RSD**	**AIC**	**BIC**	**Delta AIC**	**Log-likelihood**	**RSD**	**AIC**	**BIC**	**Delta AIC**	**Log-likelihood**
Weight										
Jenss-Bayley	0.46	5251	5300		-2613	0.46	5452	5503		-2714
Reed	0.52	5387	5424	136	-2684	0.51	5581	5619	129	-2781
Adapted Gompertz	0.49	5368	5405	117	-2675	0.49	5587	5625	135	-2784
Length/Height										
Jenss-Bayley	1.90	11786	11823		-5884	2.00	12711	12749		-6347
Reed	2.00	11794	11831	08	-5888	2.10	12739	12777	28	-6360
Adapted Gompertz	1.95	11792	11829	06	-5887	2.10	12743	12781	32	-6362

RSD = Residual standard deviation, AIC = Akaike information criterion, BIC= Bayesian information criterion

For peer review only

For peer review only

Using the Jenss-Bayley model weight and height were calculated for all the children at age points particularly when they were not measured, as well as instantaneous growth velocities at different age points, and growth trajectories were calculated (Table 4, 5 and figure 3 respectively).

Table 4 Estimated weight and weight growth velocities (SD) of girls and boys aged 0 to 6 years, from the Jenss-Bayley model

	**Boys (n = 461)**		**Girls (n = 500)**	
**Age**	**Weight, kg**	**Weight Velocity, kg/month**	**Weight, kg**	**Weight Velocity, kg/month**
3 mo	5.6 (0.6)	0.64 (0.09)	5.2 (0.6)	0.57 (0.58)
6 mo	7.1 (0.8)	0.40 (0.05)	6.6 (0.7)	0. 38 (0.05)
12 mo	8.8 (0.8)	0.30 (0.03)	8.3 (0.8)	0.21 (0.03)
2 yr	10.6 (0.9)	0.14 (0.02)	10.2 (0.9)	0.12 (0.02)
4 yr	13.7 (1.2)	0.12 (0.02)	13.3 (1.2)	0.12 (0.02)
6 yr	16.6 (1.6)	0.12 (0.02)	16.2 (1.7)	0.12 (0.02)

Table 5 Estimated length/height and length/height growth velocity (SD) of girls and boys aged 0 to 6 years, from the Jenss-Bayley model

	**Boys (n = 461)**		**Girls (n = 500)**	
Age	Length/Height, cm	Length/Height Velocity, cm/month	Length/Height, cm	Length/Height Velocity, cm/month
3 mo	59.9 (1.4)	2.67 (0.10)	58.6 (1.4)	2.55 (0.16)
6 mo	66.3 (2.8)	1.66 (0.06)	64.8 (3.0)	1.66 (0.08)
12 mo	73.4 (2.9)	0.91 (0.06)	72.1 (1.9)	0.92 (0.06)
2 yr	82.6 (3.3)	0.63 (0.05)	80.9 (2.1)	0.65 (0.04)
4 yr	96.6 (3.0)	0.61 (0.05)	95.9 (2.9)	0.62 (0.04)
6 yr	111.24 (4.1)	0.61 (0.05)	110.7 (3.7)	0.62 (0.04)

For peer review only

Means and standard deviations (SD) of parameters estimates of the Jenss-Bayley model along with two other candidate models (the adapted Gompertz model and the Reed model) fitted to weight and height of boys and girls are reported in Supplementary Table 3.

Nutritional status for all children was estimated at six years. About 1/3 of the children were wasted and underweight (Supplementary Table 4). While 8% of boys and 4% of girls were stunted (Supplementary Table 4). In comparison, in the WHO growth reference population, the percentage of children under -2 Z-scores is expected to be 2.3%.

DISCUSSION

Three growth models (the Jenss, the Reed, and an adaptation of the Gompertz model) were fitted to the weight and height of children from birth to around six years of age and their performance was compared. There was no strong trend in the residual distribution of the models over age suggesting that all the models performed well without systematic underestimation or overestimation of growth at any age. Comparison of AIC/BIC values and RSD supported the conclusion that the Jenss-Bayley model had the best fit. Also, the difference between the AIC (delta AIC) of the Jenss-Bayley model (best model) and other two candidate models demonstrated quantifiable evidence that the Jenss-Bayley model was the best one fitting on weight and height both for girls and boys[38]. Therefore, the Jenss-Bayley model was chosen to estimate mean growth trajectories and instantaneous growth velocities of children and estimated their nutritional status at 6 years.

This paper extends the previous limited studies that either fitted the Jenss-Bayley model or compared its goodness of fit with other growth models in childhood in SSA. Pagazey and Hauspie[6] successfully fitted the Jenss-Bayley model on Congolese babies weight data. However, the phase of growth studied in this study was restricted around birth to two years of age. Similarly, few studies reported Reed model fitted well. Simondon and

For peer review only

colleagues[7] compared several models on the growth data of 95 Congolese children and reported that the Reed model had a better fit but this study did not include the Jenss-Bayley and the adapted Gompertz models and studied child growth only during infancy. Chirwa and colleagues[14] reported that the Reed model fitted well on the growth data from birth to 10 years in 453 children living in an urban setting in South Africa. The study reported that the Jenss-Bayley model did not fit well in the early years. However, this study included children from an urban setting and did not test the performance of the Gompertz model. In summary, the above previous studies from SSA[6, 7, 14] either tested growth models during a limited phase of growth in early childhood or studied children only from an urban setting. The current study differs from the aforementioned studies in some respects. First, it included growth data of both infancy and early childhood. Second, it included children from a semi-rural setting which have not been studied before very well in SSA. Third, the growth data used in this study were taken from a prospective study which had standardized measurements of weight and height; therefore, it is likely to be more accurate than data from routine surveys or health records. A limitation of this study is lesser measurements between 3 and 5 years of age but as during this period weight and height are known to increase linearly with age, the impact on the obtained growth modelling parameters is likely to be less. Another limitation of this study is the generalizability of the results. These children lived in a semi-rural setting with a high prevalence of undernutrition. They also presented a high prevalence of potential risk factors for altered growth[39] (**Supplementary Table 2**). It is therefore possible that their growth would differ from a setting with different characteristics.

The estimated instantaneous growth velocities reflected the overall expected pattern of growth velocity in children i.e. a maximum during the earliest age then a decrease until two years and a constant velocity afterwards as also reported in other population of children[10, 40], although growth rate could differ depending on the population e.g. weight velocity

For peer review only

among boys at 3 years (mean +/- SD): 0.19 ± 0.06 kg/month in American children[40] vs. 0.16 ± 0.04 kg/month in French children respectively[10] and weight velocity among boys at 4 years: 0.17 ± 0.02 kg/month in South African children[41]. However, the study by Regnault et al. excluded birth weight from their analyses while the birth weight was included in the current study which could have underestimated weight growth velocities during infancy in our population[28]. The mean height growth trajectories were relatively parallel with no visible difference in the direction of the curve between boys and girls. On the contrary, visible difference in direction was observable in the mean weight growth trajectory of boys and girls. This difference was visible after infancy where the trajectory of girls falls below the curve of boys.

In general, the three models (Jenss-Bayley, adapted Gompertz and the Reed model) seemed to fit well both on weight and height data as was evidenced by mean residuals close to zero (Figure 1 and Figure 2). Certain factors could influence the models' goodness of fit when comparing several models. For example, a temporary delay to reach the maximum growth velocity after birth might cause difficulty in modelling weight by models like the Jenss-Bayley model that do not consider this pattern. The adapted Gompertz model was tested as a potential way to tackle this issue but our adaptation did not perform better than the Jenss-Bayley model. Further analysis was done to show whether predictions by the three models (Jenss-Bayley, Reed and adapted Gompertz models) at different age points i.e. at 3 months, 1 year, 3 years, and 6 years were different. The predictions differ mainly at 3 months with no major differences afterwards and the goodness of fit indicators supported the use of Jenss-Bayley model.

There were slight variations in the way the models converged. The Jenss-Bayley model converged easily on weight data using an unstructured variance-covariance matrix for random effects. More convergence issues were faced after attempting to converge the Jenss-

For peer review only

Bayley model on the height data when the number of iterations was extended to 20000. The Reed model and the adapted Gompertz model also had convergence issues both on weight and height data with an unstructured variance-covariance matrix but were able to converge with a diagonal variance-covariance matrix for random effects. Although an unstructured variance-covariance matrix would have been preferred for all the models, there was no difference in the fit indicators (i.e. AIC/BIC and RSD), fixed effects and predictions (data not shown) when using a diagonal variance-covariance matrix on the Jenss-Bayley model. In particular, a diagonal variance-covariance matrix for random effects does not constraint the growth parameters to be uncorrelated, the four model parameters being free to vary for each individual.

Convergence issues could be affected by several reasons including but not limited to the number of measurement occasions and how the time intervals between measurement occasions are spaced, complexity of the model (e.g. the number of parameters, monotonicity), parameterization (e.g. addition of higher-order terms such as ln (age))[14], as well as the type of statistical packages. Simpler methods than SAEMIX could have been used, but for consistency in methods, it was preferred to fit them with the same one. Some computational and convergence issues we faced could be explained by the limited measurement occasions and unequally spaced time intervals between 2 and 6 years as also reported by a previous study in South-Africa[14]. To facilitate the convergence of the models, constraints of positivity on the parameters of all three models was applied by using exponential functions, as shown in equation 2 for the Jenss-Bayley model[28].

CONCLUSION

This paper demonstrated that the Jenss-Bayley model presented the best fit amongst the three candidate models applied to longitudinal weight and height data of Beninese children between birth and six years. This model was then used to estimate growth trajectories

For peer review only

and growth velocities even for children with few measurements. Finally, it was estimated that about 1/3 of the children were wasted and underweight at six years of age. Parameters of this model will be used to study the determinants of growth trajectories in children.

Acknowledgements The authors gratefully acknowledge families enrolled in the above-mentioned studies and the contributors of the research team and staff.

Contributors SA, JB, and FBL collaborated in the conduct, analysis, interpretation of data, and writing. SA performed growth modelling under the supervision of JB. FBL, RZ, AG, DC, MC, AM, JA, NF contributed towards data collection and acquisition; follow up of studies; revision of the paper; and providing technical support. FBL and JB collaborated in the conception, planning, methodology, overall supervision and critical revision of the work. All authors read and approved the final manuscript.

Funding The MiPPAD study was co-funded by the European and Developing Countries Clinical Trials Partnership (EDCTP; IP.2007.31080.002), the Malaria in Pregnancy Consortium and the following national agencies: Instituto de Salud Carlos III (PI08/0564), Spain; Federal Ministry of Education and Research (BMBF FKZ: da01KA0803), Germany; Institut de Recherche pour le Developpement (IRD), France. The "Anaemia in Pregnancy: Etiologies and Consequences" (APEC) study received funding from a grant from the Bill and Melinda Gates Foundation. The TOLIMMUNPAL project was funded by Agence Nationale de la Recherche (ANR). The EXPLORE study was funded by Fondation de France. Shukrullah Ahmadi received funding from a grant from Fondation de France (reference 00079816).

Patient consent Obtained

For peer review only

Competing interest The authors declare no conflict of interest. The funders had no role in the design of the study; in the collection, analyses, or interpretation of data; in the writing of the manuscript and in the decision to publish the results.

Data sharing statement No additional data are available.

Ethics approval The MiPPAD trial was approved by the institutional review board of the University of Abomey-Calavi, Benin. The APEC study was approved by the Ethics Committee of the Faculty of Medicine of Cotonou, Benin. The TOLIMMUNPAL study was approved by the Comité Consultatif de Déontologie et d'Éthique (CCDE) of the Institut de Recherche pour le Développement (IRD, France) and the Ethics Committee of the Faculté des Sciences de la Santé, Université d'Abomey Calavi, Benin (approval number: 43/11/2010/CE/FSS/UAC). The EXPLORE study was approved by the Research Ethics Committee of Institute of Applied Biomedical Sciences, Benin (Comité d'Ethique de la Recherche, Institut des Sciences Biomédicales Appliquées, CER-ISBA, Benin) (approval number: 87 du 17/05/2016).

For peer review only

REFERENCES

1. GBD 2016 Risk Factor Collaborators. *Global, regional, and national comparative risk assessment of 84 behavioural, environmental and occupational, and metabolic risks or clusters of risks, 1990–2016: a systematic analysis for the Global Burden of Disease Study 2016.* The Lancet, 2017. **390**(10100): p. 1345-1422.
2. Berkey, C.S., *Comparison of two longitudinal growth models for preschool children.* Biometrics, 1982. **38**(1): p. 221-34.
3. Scherdel, P., et al., *Growth monitoring as an early detection tool: a systematic review.* . The Lancet Diabetes & Endocrinology 2016. **5**(4): p. 447-56.

4. Kramer, M.S., et al., *Growth During Infancy and Early Childhood and Its Association with Metabolic Risk Biomarkers at 11.5 Years.* Am J Epidemiol, 2019.

5. Regnault, N. and M.W. Gillman, *Importance of characterizing growth trajectories.* Ann Nutr Metab, 2014. **65**(2-3): p. 110-3.

6. Pagezy, H. and R. Hauspie, *Growth in weight of African babies, aged 0–24 months, living in a rural area at the Lake Tumba, Zaire.* Annals of Tropical Paediatrics, 1985. **5**(1): p. 41-47.

7. Simondon, K.B., et al., *Comparative study of five growth models applied to weight data from congolese infants between birth and 13 months of age.* Am J Hum Biol, 1992. **4**(3): p. 327-335.

8. Deming, J., *Application of the Gompertz curve to the observed pattern of growth in length of 48 individual boys and girls during the adolescent cycle of growth.* Hum Biol, 1957. **29**(1): p. 83-122.

9. Laird, A.K., *Evolution of the human growth curve.* Growth, 1967. **31**(4): p. 345-55.

10. Botton, J., et al., *Postnatal weight and height growth velocities at different ages between birth and 5 y and body composition in adolescent boys and girls.* Am J Clin Nutr, 2008. **87**(6): p. 1760-8.

11. Molinari, L. and T. Gasser, *The human growth curve: distance, velocity and acceleration. In: Hauspie, R C; Cameron, N; Molinari, L. Methods in Human Growth Research.* 2004, Cambridge University Press: Cambridge UK. p. 27-54.

12. Hauspie, R.C., Cameron, N. & Molinari, L. (eds.), *Methods in Human Growth Research*. 2004: Cambridge Press.

13. Olusanya, B.O. and J.K. Renner, *Predictors of growth velocity in early infancy in a resource-poor setting.* Early Hum Dev, 2011. **87**(10): p. 647-52.

For peer review only

14. Chirwa, E.D., et al., *Multi-level modelling of longitudinal child growth data from the Birth-to-Twenty Cohort: a comparison of growth models.* Ann Hum Biol, 2014. **41**(2): p. 168-79.

15. Singer, J.D. and J.B. Willett, *Applied longitudinal data analysis: modeling change and event occurence*. 2003, New York: Oxford University Press

16. Cameron, N., et al., *Timing and magnitude of adolescent growth in height and weight in Cape coloured children after kwashiorkor.* J Pediatr, 1986. **109**(3): p. 548-55.

17. Rozzi, F.V., et al., *Growth pattern from birth to adulthood in African pygmies of known age.* Nat Commun, 2015. **6**: p. 7672.

18. Padonou, G., et al., *Factors associated with growth patterns from birth to 18 months in a Beninese cohort of children.* Acta Trop, 2014. **135**: p. 1-9.

19. Gonzalez, R., et al., *Intermittent preventive treatment of malaria in pregnancy with mefloquine in HIV-negative women: a multicentre randomized controlled trial.* PLoS Med, 2014. **11**(9): p. e1001733.

20. Ouedraogo, S., et al., *Maternal anemia at first antenatal visit: prevalence and risk factors in a malaria-endemic area in Benin.* Am J Trop Med Hyg, 2012. **87**(3): p. 418-24.

21. d'Almeida, T.C., et al., *Soluble human leukocyte antigen -G during pregnancy and infancy in Benin: Mother/child resemblance and association with the risk of malaria infection and low birth weight.* PLoS One, 2017. **12**(2): p. e0171117.

22. Mireku, M.O., et al., *Prenatal Hemoglobin Levels and Early Cognitive and Motor Functions of One-Year-Old Children.* Pediatrics, 2015. **136**(1): p. e76-83.

23. Ahmadi, S., et al., *Hunting, Sale, and Consumption of Bushmeat Killed by Lead-Based Ammunition in Benin.* Int J Environ Res Public Health, 2018. **15**(6).

For peer review only

24. Bodeau-Livinec, F., et al., *Neurocognitive testing in West African children 3-6 years of age: Challenges and implications for data analyses.* Brain Research Bulletin, 2018.

25. World Health Organization (WHO). *Physical Status: the use and interpretation of anthropometry. A report of a WHO Expert Committee.* , in *WHO Technical Series, Geneva.* 1995, WHO.

26. World Health Organization (WHO). *The WHO Child Growth Standards*. 2006 [cited 2019.

27. Jenss, R.M. and N. Bayley, *A mathematical method for studying the growth of a child.* Human Biology, 1937. **9**(4): p. 556.

28. Botton, J., et al., *Postnatal weight and height growth modeling and prediction of body mass index as a function of time for the study of growth determinants.* Ann Nutr Metab, 2014. **65**(2-3): p. 156-66.

29. Benjamin, G., *On the nature of the function expressive of the law of human mortality, and ona new mode of determining the value of life contingencies.* . Philosophical Transactions of the Royal Society of London B: Biological Sciences, 1825: p. 513-585.

30. Karkach, A., *Trajectories and models of individual growth.* Demographic Research, 2006. **15**: p. 347-400.

31. Tjørve, K.M.C. and E. Tjørve, *The use of Gompertz models in growth analyses, and new Gompertz-model approach: An addition to the Unified-Richards family.* PLOS ONE, 2017. **12**(6): p. e0178691.

32. Winsor, C.P., *The Gompertz Curve as a Growth Curve.* Proc Natl Acad Sci U S A, 1932. **18**(1): p. 1-8.

33. Berkey, C.S. and R.B. Reed, *A model for describing normal and abnormal growth in early childhood.* Hum Biol, 1987. **59**(6): p. 973-87.

For peer review only

34. Hauspie, R.C., *Mathematical models for the study of individual growth patterns.* Rev Epidemiol Sante Publique, 1989. **37**(5-6): p. 461-76.

35. Team, R.D.C., *R: A language and environment for statistical computing.* . 2017, R Foundation for Statistical Computing: Vienna, Austria.

36. Comets, E., A. Lavenu, and M. Lavielle, *Parameter Estimation in Nonlinear Mixed Effect Models Using saemix, an R Implementation of the SAEM Algorithm.* Journal of Statistical Software, 2017. **80**(3): p. 1-42.

37. World Health Organization (WHO). *The WHO Child Growth Standards*. 2006 [cited 2019 2019 02 February]; Available from: https://www.who.int/childgrowth/standards/en/.

38. Anderson, C., et al., *Comparing predictive abilities of longitudinal child growth models.* Stat Med, 2018.

39. Accrombessi, M., et al., *Malaria in Pregnancy Is a Predictor of Infant Haemoglobin Concentrations during the First Year of Life in Benin, West Africa.* PLoS One, 2015. **10**(6): p. e0129510.

40. Regnault, N., et al., *Comparative Study of Four Growth Models Applied to Weight and Height Growth Data in a Cohort of US Children from Birth to 9 Years.* Annals of Nutrition and Metabolism, 2014. **65**(2-3): p. 167-174.

41. Chirwa, E.D., et al., *Postnatal growth velocity and overweight in early adolescents: a comparison of rural and urban African boys and girls.* Am J Hum Biol, 2014. **26**(5): p. 643-51.

FIGURE LEGENDS

Figure 1 Residuals of weight models for boys and girls

(Data of the first two deciles was gathered, as there were more than 10% of the data at birth)

For peer review only

Figure 2 Residuals of height models for boys and girls

(Data of the first two deciles was gathered, as there were more than 10% of the data at birth)

Figure 3 Weight and height mean growth trajectories from the Jenss-Bayley model. (A) Weight growth curve. (B) Height growth curve. The solid (black) line implies boys curve and dashed line implies girls

Supplementary Figure 1 Population flowchart: Follow-up until 6 years of age and showing how the final sample has been selected for growth modelling

For peer review only

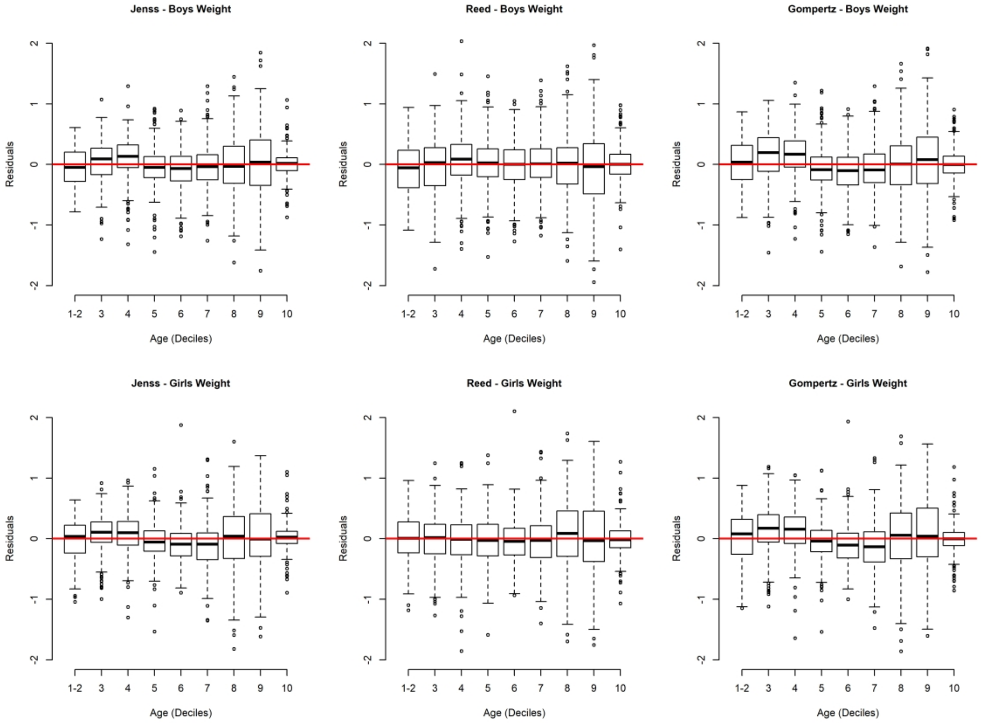

Figure 1 Residuals of weight models for boys and girls
(Data of the first two deciles was gathered, as there were more than 10% of the data at birth)

270x202mm (400 x 400 DPI)

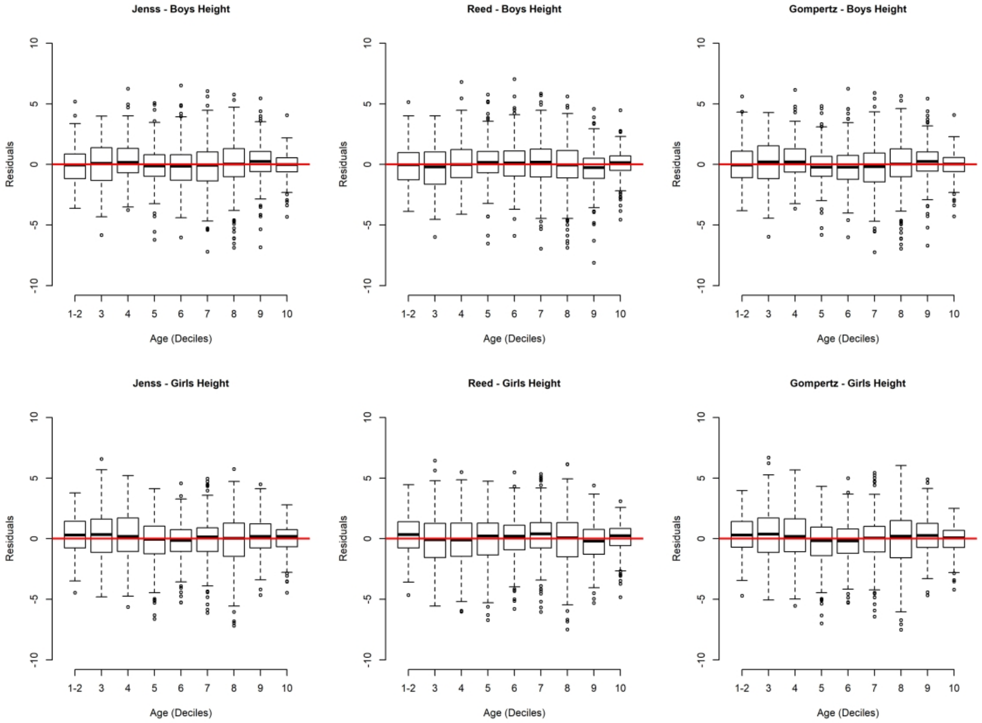

Figure 2 Residuals of height models for boys and girls
(Data of the first two deciles was gathered, as there were more than 10% of the data at birth)

270x202mm (400 x 400 DPI)

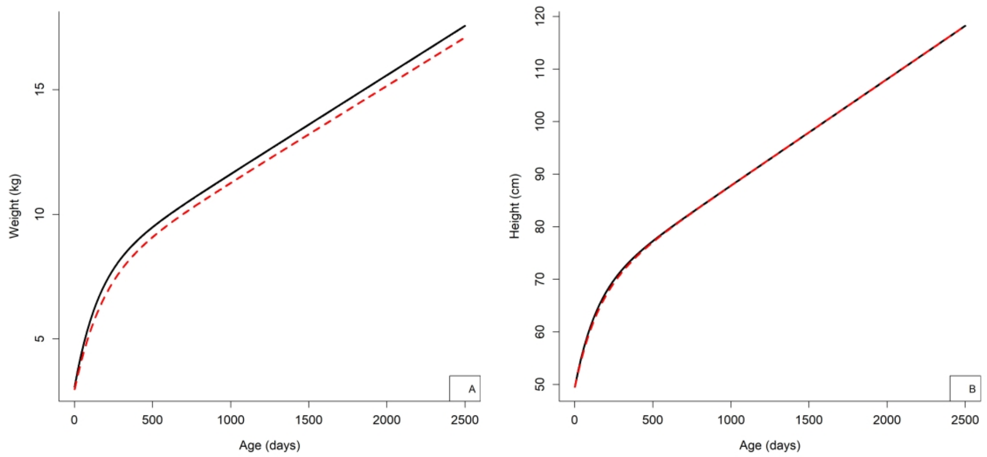

Figure 3 Weight and height mean growth trajectories from the Jenss-Bayley model. (A) Weight growth curve. (B) Height growth curve. The solid (black) line implies boys curve and dashed line implies girls

399x202mm (300 x 300 DPI)

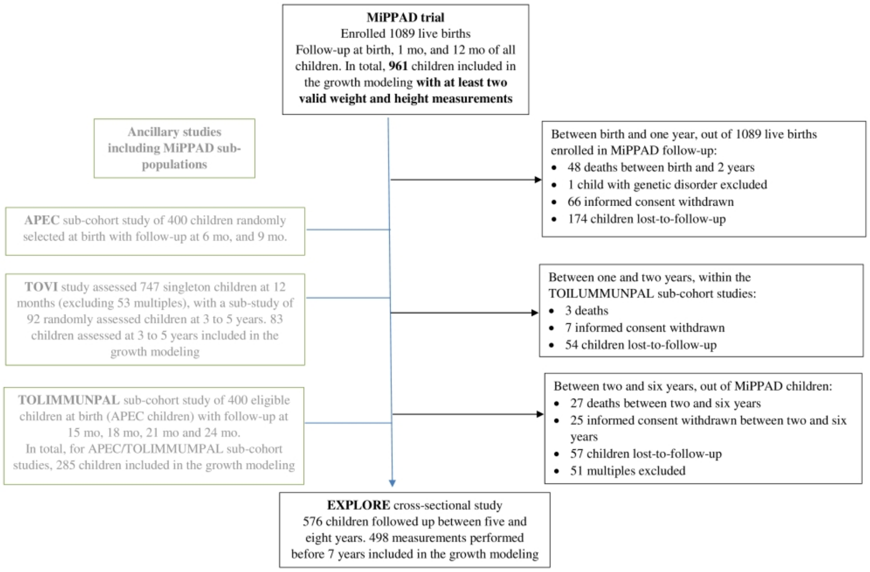

Supplementary Figure 1. Population flowchart: Follow-up until 6 years of age and showing how the final sample has been selected for growth modeling

297x210mm (300 x 300 DPI)

Supplementary tables

Supplementary Table 1. Number of children with anthropometric measurements at different age intervals. Data from boys (n=461) and girls (n=500) from birth to around six years of age

Age group	Boys	Girls
	n	n
0 – 1 yr	461	499
1 - 2 yr	196	219
2 - 3 yr	97	115
3 - 4 yr	8	10
4 - 5 yr	41	24
5 – 7 yr	253	249

Supplementary Table 2. Summary of maternal and child characteristics in the prospective child cohort in Benin, Sub-Saharan Africa.

	**Boys (n= 461)**		**Girls (n=500)**	
	**n/N**	**%**	**n/N**	**%**
Center				
Allada	51/371	13.8	49/400	12.3
Attogon	83/371	22.4	104/400	26.0
Sékou	237/371	62.9	247/400	61.8
Maternal characteristics				
Mean maternal age at the start of follow up (yr)	451/461	26.0 +/- 5.4	490/500	25.6 +/- 5.5
Maternal education [a]	112/451	24.8	106/490	21.6

For peer review only - http://bmjopen.bmj.com/site/about/guidelines.xhtml

Supplementary table 2. continued

Estimated maternal pre-pregnancy BMI (kg/m^2)				
Underweight (<18.5)	61/373	16.4	67/400	16.8
Normal (18.5 – 24.9)	262/373	70.2	298/400	74.5
Overweight (25-29.9)	35/373	9.4	26/400	6.5
Obese (≥ 30)	15/373	4.02	9/400	2.3
Gravidity				
Multigravida	305/373	81.8	326/400	81.5
Primigravida	68/373	18.2	74/400	18.5
Child characteristics				
Low birth weight (<2.5 kg)	39/440	8.9	66/481	13.7
Preterm	28/443	6.3	33/484	6.8
Malaria at 1 year	35/351	10.0	38/348	10.9
Anemia at 1 year (Hb≤110 g/L)	261/354	73.7	233/348	66.9

[a] Defined as the ability to read or write

For peer review only - http://bmjopen.bmj.com/site/about/guidelines.xhtml

Supplementary Table 3. Parameters estimates for three candidate models [a] fitted to weight and height

Model	Parameters	Boys		Girls	
		Mean	SD	Mean	SD
Weight models					
Jenss					
	A	1.11	0.05	1.08	0.06
	B	5.53	0.15	5.55	0.18
	C	1.52	0.16	1.49	0.15
	D	4.97	0.24	5.15	0.37
Adapted Gompertz	A	2.02	0.08	1.96	0.07
	B	0.90	<0.01	0.85	< 0.01
	C	4.67	0.24	4.85	0.25
	D	5.50	0.13	5.49	0.15
Reed	A	-1.18	<0.01	-0.89	0.12
	B	-5.87	0.18	-5.78	0.18
	C	0.39	0.08	0.28	0.07
	D	1.44	<0.01	1.34	<0.01
Height models					
Jenss					
	A	3.90	0.02	3.90	0.02
	B	3.90	0.08	3.90	0.06
	C	2.90	0.01	2.90	0.06
	D	4.90	0.09	5.00	0.07

For peer review only - http://bmjopen.bmj.com/site/about/guidelines.xhtml

**Supplementary table 3. continued**					
Adapted Gompertz	A	4.21	0.02	4.2	0.02
	B	0.31	< 0.01	0.3	<0.01
	C	4.83	0.13	4.9	0.12
	D	3.92	0.07	3.9	0.06
Reed	A	3.50	0.03	3.5	0.03
	B	-4.20	0.09	-4.2	0.08
	C	1.80	0.02	1.7	0.02
	D	2.80	< 0.01	2.8	<0.01

[a] the sign of the parameters for the adapted Gompertz and the Reed model was constrained by using exponential functions, as shown in equation 2 for the Jenss-Bayley model.

Supplementary Table 4. Stunting, underweight and wasting in children using predicted data from the Jenss-Bayley model. n (%)

Sex	Underweight (WAZ<–2)	Stunting (HAZ<-2)	Wasting (WHZ<-2)
Boys (n=461)	161/461 (35)	37/461 (8)	125/451 (27.7)
Girls (n=500)	168/500 (34)	22/500 (4)	190/495 (38.4)

For peer review only

BMJ Open

Comparison of Growth Models to Describe Growth from Birth to 6 Years in a Beninese Cohort of Children with Repeated Measurements

Journal:	*BMJ Open*
Manuscript ID	bmjopen-2019-035785.R2
Article Type:	Original research
Date Submitted by the Author:	30-Jun-2020

Complete List of Authors:	AHMADI, Shukrullah; INSERM, INRA, Université de Paris, Centre of Research in Epidemiology and Statistics /CRESS<br>Bodeau-Livinec, Florence; EHESP; INSERM, INRA, Université de Paris, Centre of Research in Epidemiology and Statistics /CRESS<br>Zoumenou, Roméo ; Institut de Recherche pour le Développement (IRD)<br>Garcia, Andre; Institut de Recherche pour le Développement (IRD), Université Paris Descartes, MERIT (Mère et Enfant Face aux Infections Tropicales)-UMR 216<br>Courtin, David; Institut de Recherche pour le Développement (IRD), Université Paris Descartes, MERIT (Mère et Enfant Face aux Infections Tropicales)-UMR 216<br>Alao, Jules; Paediatric Department, Mother and Child University and Hospital Center (CHU-MEL)<br>Fievet, Nadine ; Institut de Recherche pour le Développement (IRD), Université Paris Descartes, MERIT (Mère et Enfant Face aux Infections Tropicales)-UMR 216<br>Cot, Michel; Institut de Recherche pour le Développement (IRD), Université Paris Descartes, MERIT (Mère et Enfant Face aux Infections Tropicales)-UMR 216<br>Massougbodji, Achille; Université d'Abomey-Calavi Faculté des Sciences de la Santé<br>Botton, Jérémie ; EPI-PHARE, Epidemiology of health products

**Primary Subject Heading**:	Epidemiology
Secondary Subject Heading:	Paediatrics, Public health, Nutrition and metabolism, Global health
Keywords:	STATISTICS & RESEARCH METHODS, EPIDEMIOLOGY, PAEDIATRICS, PUBLIC HEALTH

SCHOLARONE™
Manuscripts

I, the Submitting Author has the right to grant and does grant on behalf of all authors of the Work (as defined in the below author licence), an exclusive licence and/or a non-exclusive licence for contributions from authors who are: i) UK Crown employees; ii) where BMJ has agreed a CC-BY licence shall apply, and/or iii) in accordance with the terms applicable for US Federal Government officers or employees acting as part of their official duties; on a worldwide, perpetual, irrevocable, royalty-free basis to BMJ Publishing Group Ltd ("BMJ") its licensees and where the relevant Journal is co-owned by BMJ to the co-owners of the Journal, to publish the Work in this journal and any other BMJ products and to exploit all rights, as set out in our licence.

The Submitting Author accepts and understands that any supply made under these terms is made by BMJ to the Submitting Author unless you are acting as an employee on behalf of your employer or a postgraduate student of an affiliated institution which is paying any applicable article publishing charge ("APC") for Open Access articles. Where the Submitting Author wishes to make the Work available on an Open Access basis (and intends to pay the relevant APC), the terms of reuse of such Open Access shall be governed by a Creative Commons licence – details of these licences and which Creative Commons licence will apply to this Work are set out in our licence referred to above.

Other than as permitted in any relevant BMJ Author's Self Archiving Policies, I confirm this Work has not been accepted for publication elsewhere, is not being considered for publication elsewhere and does not duplicate material already published. I confirm all authors consent to publication of this Work and authorise the granting of this licence.

For peer review only

Comparison of Growth Models to Describe Growth from Birth to 6 Years in a Beninese Cohort of Children with Repeated Measurements

Ahmadi S [1], Bodeau-Livinec F [1,2], Zoumenou R[3], Garcia A[4], Courtin D[4], Jules A[5], Fievet N[4], Cot M[4], Massougbodji A[6], Botton J[7].

[1] Université de Paris, Centre of Research in Epidemiology and Statistics /CRESS, INSERM, INRA, F-75004, Paris, France. shukrullah.ahmadi@inserm.fr
[2] EHESP, F-35000 Rennes, France. florence.bodeau-livinec@ehesp.fr
[3] Institut de Recherche pour le Développement (IRD), Cotonou, Benin. zoumenour@yahoo.fr
[4] MERIT (Mère et Enfant Face aux Infections Tropicales) - UMR 216, Institut de Recherche pour le Développement (IRD), Université Paris Descartes, 75006 Paris, France. andre.garcia@ird.fr; david.courtin@ird.fr; nadine.fievet@ird.fr; michel.cot@ird.fr
[5] Paediatric Department, Mother and Child University and Hospital Center (CHU-MEL), Cotonou, Benin. amomj@yahoo.fr
[6] Faculté des Sciences de la Santé, Université d'Abomey-Calavi, Cotonou, Benin. massougbodjiachille@yahoo.fr
[7] EPI-PHARE, Epidemiology of health products, Saint-Denis, France. jeremie.botton@ansm.sante.fr

Correspondence to Shukrullah Ahmadi, Centre of Research in Epidemiology and Statistics /CRESS. INSERM, INRA, F-75004, Paris, France. shukrullah.ahmadi@inserm.fr

Word count: ≈ 3900

For peer review only - http://bmjopen.bmj.com/site/about/guidelines.xhtml

ABSTRACT

Objective To select a growth model that best describes individual growth trajectories of children and to present some growth characteristics of this population.

Settings Participants were selected from a prospective cohort conducted in three health centres (Allada, Sekou and Attogon) in a semi-rural region of Benin, Sub-saharan, Africa.

Participants Children aged 0-6 years recruited in a cohort study with at least two valid height and weight measurements were included (n=961).

Primary and secondary outcome measures This study compared the goodness of fit of three structural growth models (Jenss-Bayley, Berkey-Reed and a newly adapted version of the Gompertz growth model) on longitudinal weight and height growth data of boys and girls. The goodness-of-fit of the models was assessed using residual distribution over age and compared with the Akaike Information Criterion (AIC) and Bayesian Information Criterion (BIC). The best-fitting model allowed estimating mean weight and height growth trajectories, individual growth and growth velocities. Underweight, stunting and wasting were also estimated at 6 years.

Results The three models were able to fit well both weight and height data. The Jenss-Bayley model presented the best fit for weight and height, both in boys and girls. Mean height growth trajectories were identical in shape and direction for boys and girls while the mean weight growth curve of girls fell slightly below the curve of boys after neonatal life. Finally, 35%, 27.7% and 8% of boys; and 34%, 38.4%, and 4% of girls were estimated to be underweight, wasted, and stunted at 6 years, respectively.

Conclusion The growth parameters of the best fitting Jenss-Bayley model can be used to describe growth trajectories and study their determinants

For peer review only

Keywords Weight, height, growth modelling, mixed-effects modelling, structural growth models, sub-Saharan Africa, Growth trajectory

Strengths and limitations of this study

- This is the first study to assess the comparative merits of growth models on child anthropometric data in a semi-rural setting in sub-Saharan Africa with a high prevalence of undernutrition.
- The generalizability of these results cannot be guaranteed because the study sample is not representative of the Beninese children.
- Other models could have been tested but the overall fit of the selected models was very optimal and they present advantages for future studies in this population.

INTRODUCTION

Insufficient growth during childhood adversely affects later health outcomes. According to estimates from the Global Burden of Diseases, Injuries, and Risk Factors Study 2016[1], the prevalence of stunting, wasting, and underweight (based on the definitions using the WHO 2006 growth standards) in children under five were, respectively, 36.6%, 8.6% and 19.5% in sub-Saharan Africa (SSA) in 2015. Furthermore, more than 23% of under-five mortality was attributable to child growth failure, being thus the second leading risk factor for child mortality in this region.

Mathematical growth modelling is a powerful tool for the study of child growth and growth trajectories. It consists of fitting models to physical growth data (e.g. weight,

For peer review only

length/height, head circumference) to obtain an appropriate growth curve that will conveniently summarize growth information provided by weight and height measurements of children, even from irregularly spaced growth measurements[2]. Clinicians routinely compare growth data to growth charts to identify impaired growth trajectories in children. Indeed, looking at growth trajectories (weight, height/length or BMI) has gained importance for several purposes. It is becoming essential for surveillance to identify abnormal growth trajectories[3]. In the post-natal period, length/height and weight surveillance is an essential tool for monitoring child growth. It is also used in research to study determinants of growth including identification of modifiable risk factors and sensitive age periods during which interventions may be especially useful to achieve optimal growth[4]. Characterizing growth trajectories is also important to study whether past growth is associated with future growth patterns or later health outcomes[5].

There are several structural and non-structural growth models to describe child growth, amongst them the Jenss-Bayley model[6], the Reed model[7], and the Gompertz functions[8, 9]. These three models are amongst the most common structural models described in the literature, while non-structural models are mainly polynomial and splines[10-13], as cited in Chirwa et al. (2014)[14]. The parameters of structural models have a biological basis. In contrast, non-structural models do not formulate any particular form of growth curve and may demonstrate instability at extremities. Besides, the parameters obtained from non-structural models do not provide any biological interpretation[12, 15].

Many studies have analysed child growth data in Africa, but few studies[6, 7, 13, 14, 16-18] have applied structural or non-structural growth models on African longitudinal growth data. Very few of these studies[7, 14] have assessed the comparative merits of models like the Jenss-Bayley and Gompertz model on child growth in SSA. Furthermore, as to our knowledge, no study has compared the fitness of the adapted Gompertz growth model on child growth data.

For peer review only

This study aimed to compare the fit of three structural growth models (the Jenss-Bayley model, the Reed model, and a new model which is an adaptation of the Gompertz model) applied to weight and height growth data in a population of Beninese children aged 0-6 years, living in a semi-rural region and to describe their growth using the selected model.

METHODOLOGY

Study design and data sources

Weight and height measurements were available from a prospective cohort study of Beninese children followed from birth to age 6. These children who participated were born within the MiPPAD clinical trial (Malaria in Pregnancy Preventive Alternative Drugs) and followed in three ancillary studies, the APEC, TOLIMMUNPAL and TOVI studies, and finally assessed in the EXPLORE study at 6 years, on average. The research objectives, design, sample size, and follow-up period varied for these studies.

Participating children were born of HIV-negative mothers (n = 1182) enrolled in the MiPPAD clinical trial (Malaria in Pregnancy Preventive Alternative Drugs) (NCT00811421) in 2011 in the district of Allada, south Benin[19]. The MiPPAD trial, which enrolled pregnant women before 28 weeks of gestation at the first antenatal care visit, compared the efficacy of two intermittent preventive treatments for malaria in pregnancy (IPTp). Height and weight of children were assessed at birth and women were requested to bring their infants to the health centre when the babies were 1 month, and also 9 and 12 months old. Children included in our analyses met the following inclusion criteria: being born within the MiPPAD clinical trial, and assessed at least twice with valid anthropometric measurements (weight and height) between birth and age 6 within MiPPAD, APEC, TOLIMMUNPAL, TOVI or EXPLORE studies.

Within this (MiPPAD) cohort, a sub-sample of 400 children was followed more closely. These children were followed from birth to 2 years in the APEC (Anemia in pregnancy: etiology

For peer review only

and consequences)[20] and TOLIMMUNPAL studies[21]. The APEC study included the first 400 offspring born within the MiPPAD study including anthropometric measurements of children at 6 and 9 months. Infants assessed in the APEC study were also followed-up in the TOLIMMUNPAL study between birth and 24 months with further anthropometric measurements at 15, 18, 21 and 24 months.

The TOVI study followed-up 747 singleton 1-year-old children born within MiPPAD. Among these children, only 92 children randomly selected were assessed between 3 and 5 years for anthropometric measurements[22]. Finally, at age 6, children were assessed within the EXPLORE study at 6 years on average for anthropometric measurements (2016 -2018)[23, 24]. Supplementary Figure 1 shows participants inclusion and how different ancillary studies are embedded in this cohort.

Appropriate participant consent and ethics approval were obtained for the different studies included.

Patient and public involvement

Patients and the public were not directly involved in the planning of this study. Results will be communicated to participants via community meetings with local leaders.

Anthropometric measurement

Measurements were performed by trained staff at each visit, with children wearing light clothes only and no shoes. From birth up to around 2 years, infants' length was measured to the nearest 1 mm using a locally manufactured wooden measuring scale according to the WHO guidelines[25]. From birth up to 2 years, infants' weight was measured using an electronic baby scale (SECA type 354) with a precision of 10 g. From 2 years onwards, standing weight and height was measured for all children. Height was measured by a locally manufactured length board. The length board comprised of a measuring board mounted to the wall with an attached

For peer review only

measuring tape and moveable right angle headpiece. Weight was measured using an analogue scale (CAMRY DT602). At 6 years, children were weighted with the Tanita scale.

Data inclusion

Participants with at least two weight or height measurements anytime between birth and around 6 years were included in the analysis. Weight-for-age z-scores (WAZ) and height-for-age z-scores (HAZ) were calculated based on the WHO growth standards[26]. To identify outliers, as the distribution of the Z-scores was shifted to the left, the distribution was corrected by centring it to the mean. Then measurements with centred WAZ or HAZ greater than +4 or less than -4 were considered outliers for growth and were systematically excluded from the analysis. Additionally, individual weight and height profiles were plotted to visually check individual growth trajectories for plausibility. 65 height (out of 5291 measures) and 35 weight observations (out of 5291 measures) were excluded. They were considered as outliers on the descriptive graph even after the systematic removal of observations with WAZ or HAZ greater than +4 or less than -4. The final analyses had 961 participants (461 boys and 500 girls). The number of children with anthropometric measurements at different age intervals is presented in the supplementary Table 1.

Growth models

The following three structural growth models were compared: the Jenss-Bayley model, the Reed model, and a new model which is an adaptation of the Gompertz model. These models are presented in the equations 1, 3 and 4, where E (y_{ij}) is the expected value (weight or length of child i at j^{th} occasion, t_{ij} is age in days, A_i, B_i C_i and D_i are the parameters of the functions for the ith child.

The Jenss-Bayley model[27] is presented in equation 1 and as another parameterization in equation 2. Briefly, a or $\exp^{A}$ is the predicted value at birth (t=0); b or $\exp^{-B}$ is the asymptotic slope or growth rate from about 2 years onwards; d or $\exp^{-D}$ allows for decelerating growth

(decreasing exponential function) during infancy. The parameter c or exp^C can reflect the degree of catch-up growth. These four parameters or model coefficients can be obtained for each individual-thanks to a mixed-effects modelling approach- and represent the global individual growth trajectory. Parameters (or a combination of them, e.g. the velocity) can then be studied in association with the determinants of growth or other independent variables[28].

$$E(y_{ij}) = a_i + b_i \cdot t_{ij} - (exp^{c_i + d_i . t_{ij}}) \quad (1)$$

$$E(y_{ij}) = exp^{A_i} + exp^{-B_i} \cdot t_{ij} + exp^{C_i} \cdot (1 - exp^{-exp^{-D_i} \cdot t_{ij}}) \quad (2)$$

The Gompertz model[29] (equation 3 with Di=0) is a 3-parameter model commonly used to model human growth and development[30, 31]. Parameter A_i represents the upper asymptote (maximum value), C_i represents growth rate and B_i is related to weight at birth (t=0). The Gompertz model theoretically approaches a null asymptote at later ages and to better represent the positive linear growth between about 3 and 6 years, the Gompertz growth function[31, 32] was expanded by adding a 4th parameter D_i on height and weight modelling. This adaptation allows the model to have a non-flat asymptote (slope=velocity different from 0). This permits to fit the linear part of the growth at the later ages in our data (constant linear growth velocity). The model is non-linear in the model parameters.

$$E(y_{ij}) = A_i \cdot exp(-B_i \cdot exp(-C_i \cdot t_{ij})) + D_i \cdot t_{ij} \quad (3)$$

In the Reed model[33], parameter B_i is the asymptotic slope or growth rate/velocity of preschool growth, C_i is an important component of rapid early childhood growth[2, 34] and models the exponential growth deceleration, D_i is used to better model early life variations in growth, and A_i is the intercept. The model is as follows:

$$E(y_{ij}) = A_i + B_i.t_{ij} + C_i.ln(t_{ij} + 1) + \frac{D_i}{t_{ij}} \quad (4)$$

For peer review only

Statistical methods

The Jenss-Bayley model, the Reed model, and the adapted Gompertz model were applied to weight and height growth data of children from birth to 6 years.

The Reed fixed-effects model was fit using the lm (linear model) function in R[35], and the Jenss and Gompertz models using the nls (Nonlinear Least Squares) function in R [35] to obtain initial values more easily[28]. Then the models were fitted to weight and height data using the SAEMIX[36] package in R-3.5.1 software[35] as applied in another study[28]. Due to convergence issues with unstructured matrices, a diagonal variance-covariance matrix was selected for random effects.

Growth trajectories of the children were estimated using a recently proposed method described elsewhere[28]. Briefly, weight and height growth trajectories and growth velocities (first derivative of the equation) was estimated using the model presenting the best fit (the Jenss-Bayley model). Fixed-effect parameters (A, B, C and D) obtained from the mixed-effects model using SAEMIX package were used to represent average growth trajectories. The Jenss-Bayley model then allowed predicting individual weight and height by substituting the individual model parameters into their corresponding model equation (equation 2). Similarly, individual growth velocities were calculated by substituting determined individual parameters into the derivative of the Jenss-Bayley model. As a derivative of the Jenss-Bayley model, the following equation can be used to estimate growth velocity over time[28].

$$dy/dt = exp^{-B_i} + exp^{C_i - D_i - exp^{-D_i \cdot t_{ij}}} \qquad (5)$$

Finally, undernutrition (underweight, stunting, and wasting) of children was estimated at 6 years using predicted data by the best-fitting model. Children with weight-for-age-z-score <-2 were classified as underweight, those with height-for-age-z-score <-2 as stunted, and those with weight-for-height-z-score <-2 were classified as wasted[37].

For peer review only

Comparison of Goodness of Fit

The three non-nested models were compared using the Akaike information criterion (AIC), delta AIC, and Bayesian information criterion (BIC). Lower AIC and BIC values indicate a better fit. The delta AIC [38] for a candidate model, is the difference between the AIC values of the best and the candidate models. This difference (delta AIC) is then used as follows to determine the amount of support for each candidate model. If the delta AIC is < 2, there is substantial evidence to support the candidate model (i.e., the candidate model is almost as best performing as the best model). If the delta AIC is between 4 and 7, there is considerably less support for the candidate model to be the best model. Finally, if the delta AIC is >10, there is essentially no support for the candidate model to be better than the best model. Additionally, residuals of the models were estimated by subtracting observed measurements (weight and height) from the model-predicted ones. The distribution of the residuals of the models was plotted against age. The residual standard deviation for each model was also reported.

RESULTS

Three models were fitted to weight and height growth data of 961 children from birth to 6 years separately for boys and girls (Table 1). Children had between two and 11 weight and height measurements (Table 1), and 75% of girls had at least four measurements (five in boys). Some descriptive characteristics of the study population are presented in supplementary table 2. The overall mean maternal age at age 1 year of the study child was 25.8 years (s.d. 5.6). Almost ¾ of mothers could not read or write at the start of the cohort. Almost 10% of mothers were underweight before their pregnancy. Almost 10% of boys and 14% of girls had a lower birth weight (<2.5 kg) and less than 7 % of children were born preterm. Almost 10% of children had malaria at 1 year of age and almost 74% of boys and 67% of girls had anaemia at 1 year of age.

Table 1 Number of anthropometric measures per child and sex

For peer review only - http://bmjopen.bmj.com/site/about/guidelines.xhtml

Gender	n	Growth dimension	Measures					
			N	Min	Q1	Median	Q3	Max
Girls	500	Weight	2680	2	4	6	9	11
	500	Length/Height	2680	2	4	6	9	11
Boys	461	Weight	2514	2	5	6	9	11
	461	Length/Height	2514	2	5	6	9	11

N: total number of measurements; Min: minimum; Q1: first quartile; Q3: 3rd quartile, Max: maximum.

The distribution of the residuals over age for weight and height of both boys and girls showed that, overall, residuals were centred around zero for all ages and there was no strong dependence of residual distribution over ages for all the models (Figure 1 and Figure 2).

For weight models, the Jenss-Bayley model had the lowest AIC and BIC values, both in boys and girls, indicating a better fit than the two other candidate models (Table 2). Both the Reed and the adapted Gompertz model had high delta AIC values for both boys (136 and 117, respectively) and girls (129 and 135, respectively) indicating essentially no support for these two candidate model to be better than the best fitting model (Jenss-Bayley).

For height models, the Jenss-Bayley model and the adapted Gompertz model had the lowest AIC and BIC values for the height model in boys while the Jenss-Bayley model had the lowest AIC values in girls than the Reed and adapted Gompertz model. Concerning delta AIC values, both the Reed and the adapted Gompertz model had values less than 10 for boys (8 and 6, respectively) indicating considerably less support for these two models to be the best fitting model. While in girls, these two candidate models had delta AIC values >10 (28 and 32, respectively) which does not indicate any support for these two models to have a better fit than the Jenss-Bayley model.

For peer review only

For peer review only

Table 2 Comparison of the goodness of fit of the three candidate models

	Boys					Girls				
**Model**	**RSD**	**AIC**	**BIC**	**Delta AIC**	**Log-likelihood**	**RSD**	**AIC**	**BIC**	**Delta AIC**	**Log-likelihood**
Weight										
Jenss-Bayley	0.46	5251	5300		-2613	0.46	5452	5503		-2714
Reed	0.52	5387	5424	136	-2684	0.51	5581	5619	129	-2781
Adapted Gompertz	0.49	5368	5405	117	-2675	0.49	5587	5625	135	-2784
Length/Height										
Jenss-Bayley	1.90	11786	11823		-5884	2.00	12711	12749		-6347
Reed	2.00	11794	11831	08	-5888	2.10	12739	12777	28	-6360
Adapted Gompertz	1.95	11792	11829	06	-5887	2.10	12743	12781	32	-6362

RSD = Residual standard deviation, AIC = Akaike information criterion, BIC= Bayesian information criterion

For peer review only

For peer review only

Using the Jenss-Bayley model weight and height were calculated for all the children at age points particularly when they were not measured, as well as instantaneous growth velocities at different age points, and growth trajectories were calculated (Table 3, 4 and figure 3 respectively).

Table 3 Estimated weight and weight growth velocities (SD) of girls and boys aged 0 to 6 years, from the Jenss-Bayley model

	**Boys (n = 461)**		**Girls (n = 500)**	
**Age**	**Weight, kg**	**Weight Velocity, kg/month**	**Weight, kg**	**Weight Velocity, kg/month**
3 mo	5.6 (0.6)	0.64 (0.09)	5.2 (0.6)	0.57 (0.58)
6 mo	7.1 (0.8)	0.40 (0.05)	6.6 (0.7)	0. 38 (0.05)
12 mo	8.8 (0.8)	0.30 (0.03)	8.3 (0.8)	0.21 (0.03)
2 yr	10.6 (0.9)	0.14 (0.02)	10.2 (0.9)	0.12 (0.02)
4 yr	13.7 (1.2)	0.12 (0.02)	13.3 (1.2)	0.12 (0.02)
6 yr	16.6 (1.6)	0.12 (0.02)	16.2 (1.7)	0.12 (0.02)

Table 4 Estimated length/height and length/height growth velocity (SD) of girls and boys aged 0 to 6 years, from the Jenss-Bayley model

	**Boys (n = 461)**		**Girls (n = 500)**	
Age	Length/Height, cm	Length/Height Velocity, cm/month	Length/Height, cm	Length/Height Velocity, cm/month
3 mo	59.9 (1.4)	2.67 (0.10)	58.6 (1.4)	2.55 (0.16)
6 mo	66.3 (2.8)	1.66 (0.06)	64.8 (3.0)	1.66 (0.08)
12 mo	73.4 (2.9)	0.91 (0.06)	72.1 (1.9)	0.92 (0.06)
2 yr	82.6 (3.3)	0.63 (0.05)	80.9 (2.1)	0.65 (0.04)
4 yr	96.6 (3.0)	0.61 (0.05)	95.9 (2.9)	0.62 (0.04)
6 yr	111.24 (4.1)	0.61 (0.05)	110.7 (3.7)	0.62 (0.04)

For peer review only

Means and standard deviations (SD) of parameters estimates of the Jenss-Bayley model along with two other candidate models (the adapted Gompertz model and the Reed model) fitted to weight and height of boys and girls are reported in Supplementary Table 3.

Nutritional status for all children was estimated at 6 years. About 1/3 of the children were wasted and underweight (Supplementary Table 4). While 8% of boys and 4% of girls were stunted (Supplementary Table 4). In comparison, in the WHO growth reference population, the percentage of children under -2 Z-scores is expected to be 2.3%.

DISCUSSION

Three growth models (the Jenss, the Reed, and an adaptation of the Gompertz model) were fitted to the weight and height of children from birth to around 6 years and their performance was compared. There was no strong trend in the residual distribution of the models over age suggesting that all the models performed well without systematic underestimation or overestimation of growth at any age. Comparison of AIC/BIC values and RSD supported the conclusion that the Jenss-Bayley model had the best fit. Also, the difference between the AIC (delta AIC) of the Jenss-Bayley model (best model) and other two candidate models demonstrated quantifiable evidence that the Jenss-Bayley model was the best one fitting on weight and height both for girls and boys[38]. Therefore, the Jenss-Bayley model was chosen to estimate mean growth trajectories and instantaneous growth velocities of children and estimated their nutritional status at 6 years.

This paper extends the previous limited studies that either fitted the Jenss-Bayley model or compared its goodness of fit with other growth models in childhood in SSA. Pagazey and Hauspie[6] successfully fitted the Jenss-Bayley model on Congolese babies weight data. However, the phase of growth studied in this study was restricted around birth to 2 years. Similarly, few studies reported Reed model fitted well. Simondon and colleagues[7] compared

For peer review only

several models on the growth data of 95 Congolese children and reported that the Reed model had a better fit but this study did not include the Jenss-Bayley and the adapted Gompertz models and studied child growth only during infancy. Chirwa and colleagues[14] also reported that the Reed model fitted well on the growth data from birth to 10 years in 453 children living in an urban setting in South Africa. The study reported that the Jenss-Bayley model did not fit well in the early years. However, this study included children only from an urban setting and did not test the performance of the Gompertz model. In summary, the above previous studies from SSA[6, 7, 14] either tested growth models during a limited phase of growth in early childhood or studied children only from an urban setting. The current study differs from the aforementioned studies in some respects. First, it included growth data of both infancy and early childhood. Second, it included children from a semi-rural setting which have not been studied before very well in SSA. Third, the growth data used in this study were taken from a prospective study which had standardized measurements of weight and height; therefore, it is likely to be more accurate than data from routine surveys or health records. A limitation of this study is lesser measurements between 3 and 5 years but as during this period weight and height are known to increase linearly with age, the impact on the obtained growth modelling parameters is likely to be less. Another limitation of this study is the generalizability of the results. These children lived in a semi-rural setting with a high prevalence of undernutrition. They also presented a high prevalence of potential risk factors for altered growth[39] (**Supplementary Table 2**). It is therefore possible that their growth would differ from a setting with different characteristics.

The estimated instantaneous growth velocities reflected the overall expected pattern of growth velocity in children i.e. a maximum during the earliest age then a decrease until 2 years and a constant velocity afterwards as also reported in other population of children[10, 40], although growth rate could differ depending on the population e.g. weight velocity among boys

For peer review only

at 3 years (mean +/- SD): 0.19 ± 0.06 kg/month in American children[40] vs. 0.16 ± 0.04 kg/month in French children respectively[10] and weight velocity among boys at 4 years: 0.17 ± 0.02 kg/month in South African children[41]. However, the study by Regnault et al. excluded birth weight from their analyses while the birth weight was included in the current study which could have underestimated weight growth velocities during infancy in our population[28]. The mean height growth trajectories were relatively parallel with no visible difference in the direction of the curve between boys and girls. On the contrary, visible difference in direction was observable in the mean weight growth trajectory of boys and girls. This difference was visible after infancy where the trajectory of girls falls below the curve of boys.

In general, the three models (Jenss-Bayley, adapted Gompertz and the Reed model) seemed to fit well both on weight and height data as was evidenced by mean residuals close to zero (Figure 1 and Figure 2). Certain factors could influence the models' goodness of fit when comparing several models. For example, a temporary delay to reach the maximum growth velocity after birth might cause difficulty in modelling weight by models like the Jenss-Bayley model that do not consider this pattern. The adapted Gompertz model was tested as a potential way to tackle this issue but our adaptation did not perform better than the Jenss-Bayley model. Further analysis was done to show whether predictions by the three models (Jenss-Bayley, Reed and adapted Gompertz models) at different age points i.e. at 3 months, 1 year, 3 years, and 6 years were different. The predictions differ mainly at 3 months with no major differences afterwards and the goodness of fit indicators supported the use of Jenss-Bayley model (data not shown).

There were slight variations in the way the models converged. The Jenss-Bayley model converged easily on weight data using an unstructured variance-covariance matrix for random effects. More convergence issues were faced after attempting to converge the Jenss-Bayley model on the height data when the number of iterations was extended to 20000. The Reed model

For peer review only

and the adapted Gompertz model also had convergence issues both on weight and height data with an unstructured variance-covariance matrix but were able to converge with a diagonal variance-covariance matrix for random effects. Although an unstructured variance-covariance matrix would have been preferred for all the models, there was no difference in the fit indicators (i.e. AIC/BIC and RSD), fixed effects and predictions (data not shown) when using a diagonal variance-covariance matrix on the Jenss-Bayley model. In particular, a diagonal variance-covariance matrix for random effects does not constraint the growth parameters to be uncorrelated, as the fixed part (population parameters) are free to be correlated anyway.

Convergence issues could be affected by several reasons including but not limited to the number of measurement occasions and how the time intervals between measurement occasions are spaced, complexity of the model (e.g. the number of parameters, monotonicity), parameterization (e.g. addition of higher-order terms such as ln (age))[14], as well as the type of statistical packages. Simpler methods than SAEMIX could have been used, but for consistency in methods, it was preferred to fit them with the same one. Some computational and convergence issues we faced could be explained by the limited measurement occasions and unequally spaced time intervals between 2 and 6 years as also reported by a previous study in South-Africa[14]. To facilitate the convergence of the models, constraints of positivity on the parameters of all three models were applied by using exponential functions, as shown in equation 2 for the Jenss-Bayley model[28].

CONCLUSION

This paper demonstrated that the Jenss-Bayley model presented the best fit amongst the three candidate models applied to longitudinal weight and height data of Beninese children between birth and 6 years. This model was then used to estimate growth trajectories and growth velocities even for children with few measurements. Finally, it was estimated that about 1/3 of

For peer review only

the children were wasted and underweight at 6 years. Parameters of this model will be used to study the determinants of growth trajectories in children.

Acknowledgements The authors gratefully acknowledge families enrolled in the above-mentioned studies and the contributors of the research team and staff.

Contributors SA, JB, and FBL collaborated in the conduct, analysis, interpretation of data, and writing. SA performed growth modelling under the supervision of JB. FBL, RZ, AG, DC, MC, AM, JA, NF contributed towards data collection and acquisition; follow up of studies; revision of the paper; and providing technical support. FBL and JB collaborated in the conception, planning, methodology, overall supervision and critical revision of the work. All authors read and approved the final manuscript.

Funding The MiPPAD study was co-funded by the European and Developing Countries Clinical Trials Partnership (EDCTP; IP.2007.31080.002), the Malaria in Pregnancy Consortium and the following national agencies: Instituto de Salud Carlos III (PI08/0564), Spain; Federal Ministry of Education and Research (BMBF FKZ: da01KA0803), Germany; Institut de Recherche pour le Developpement (IRD), France. The "Anaemia in Pregnancy: Etiologies and Consequences" (APEC) study received funding from a grant from the Bill and Melinda Gates Foundation. The TOLIMMUNPAL project was funded by Agence Nationale de la Recherche (ANR). The EXPLORE study was funded by Fondation de France. Shukrullah Ahmadi received funding from a grant from Fondation de France (reference 00079816).

Patient consent Obtained

Competing interest The authors declare no conflict of interest. The funders had no role in the design of the study; in the collection, analyses, or interpretation of data; in the writing of the manuscript and in the decision to publish the results.

Data sharing statement No additional data are available.

For peer review only

Ethics approval The MiPPAD trial was approved by the institutional review board of the University of Abomey-Calavi, Benin. The APEC study was approved by the Ethics Committee of the Faculty of Medicine of Cotonou, Benin. The TOLIMMUNPAL study was approved by the Comité Consultatif de Déontologie et d'Éthique (CCDE) of the Institut de Recherche pour le Développement (IRD, France) and the Ethics Committee of the Faculté des Sciences de la Santé, Université d'Abomey Calavi, Benin (approval number: 43/11/2010/CE/FSS/UAC). The EXPLORE study was approved by the Research Ethics Committee of Institute of Applied Biomedical Sciences, Benin (Comité d'Ethique de la Recherche, Institut des Sciences Biomédicales Appliquées, CER-ISBA, Benin) (approval number: 87 du 17/05/2016).

REFERENCES

1. GBD 2016 Risk Factor Collaborators. *Global, regional, and national comparative risk assessment of 84 behavioural, environmental and occupational, and metabolic risks or clusters of risks, 1990–2016: a systematic analysis for the Global Burden of Disease Study 2016.* The Lancet, 2017. **390**(10100): p. 1345-1422.
2. Berkey, C.S., *Comparison of two longitudinal growth models for preschool children.* Biometrics, 1982. **38**(1): p. 221-34.
3. Scherdel, P., et al., *Growth monitoring as an early detection tool: a systematic review.* . The Lancet Diabetes & Endocrinology 2016. **5**(4): p. 447-56.
4. Kramer, M.S., et al., *Growth During Infancy and Early Childhood and Its Association with Metabolic Risk Biomarkers at 11.5 Years.* Am J Epidemiol, 2019.
5. Regnault, N. and M.W. Gillman, *Importance of characterizing growth trajectories.* Ann Nutr Metab, 2014. **65**(2-3): p. 110-3.
6. Pagezy, H. and R. Hauspie, *Growth in weight of African babies, aged 0–24 months, living in a rural area at the Lake Tumba, Zaire.* Annals of Tropical Paediatrics, 1985. **5**(1): p. 41-47.

For peer review only

7. Simondon, K.B., et al., *Comparative study of five growth models applied to weight data from congolese infants between birth and 13 months of age.* Am J Hum Biol, 1992. **4**(3): p. 327-335.

8. Deming, J., *Application of the Gompertz curve to the observed pattern of growth in length of 48 individual boys and girls during the adolescent cycle of growth.* Hum Biol, 1957. **29**(1): p. 83-122.

9. Laird, A.K., *Evolution of the human growth curve.* Growth, 1967. **31**(4): p. 345-55.

10. Botton, J., et al., *Postnatal weight and height growth velocities at different ages between birth and 5 y and body composition in adolescent boys and girls.* Am J Clin Nutr, 2008. **87**(6): p. 1760-8.

11. Molinari, L. and T. Gasser, *The human growth curve: distance, velocity and acceleration. In: Hauspie, R C; Cameron, N; Molinari, L. Methods in Human Growth Research.* 2004, Cambridge University Press: Cambridge UK. p. 27-54.

12. Hauspie, R.C., Cameron, N. & Molinari, L. (eds.), *Methods in Human Growth Research*. 2004: Cambridge Press.

13. Olusanya, B.O. and J.K. Renner, *Predictors of growth velocity in early infancy in a resource-poor setting.* Early Hum Dev, 2011. **87**(10): p. 647-52.

14. Chirwa, E.D., et al., *Multi-level modelling of longitudinal child growth data from the Birth-to-Twenty Cohort: a comparison of growth models.* Ann Hum Biol, 2014. **41**(2): p. 168-79.

15. Singer, J.D. and J.B. Willett, *Applied longitudinal data analysis: modeling change and event occurence*. 2003, New York: Oxford University Press

16. Cameron, N., et al., *Timing and magnitude of adolescent growth in height and weight in Cape coloured children after kwashiorkor.* J Pediatr, 1986. **109**(3): p. 548-55.

For peer review only

17. Rozzi, F.V., et al., *Growth pattern from birth to adulthood in African pygmies of known age.* Nat Commun, 2015. **6**: p. 7672.

18. Padonou, G., et al., *Factors associated with growth patterns from birth to 18 months in a Beninese cohort of children.* Acta Trop, 2014. **135**: p. 1-9.

19. Gonzalez, R., et al., *Intermittent preventive treatment of malaria in pregnancy with mefloquine in HIV-negative women: a multicentre randomized controlled trial.* PLoS Med, 2014. **11**(9): p. e1001733.

20. Ouedraogo, S., et al., *Maternal anemia at first antenatal visit: prevalence and risk factors in a malaria-endemic area in Benin.* Am J Trop Med Hyg, 2012. **87**(3): p. 418-24.

21. d'Almeida, T.C., et al., *Soluble human leukocyte antigen -G during pregnancy and infancy in Benin: Mother/child resemblance and association with the risk of malaria infection and low birth weight.* PLoS One, 2017. **12**(2): p. e0171117.

22. Mireku, M.O., et al., *Prenatal Hemoglobin Levels and Early Cognitive and Motor Functions of One-Year-Old Children.* Pediatrics, 2015. **136**(1): p. e76-83.

23. Ahmadi, S., et al., *Hunting, Sale, and Consumption of Bushmeat Killed by Lead-Based Ammunition in Benin.* Int J Environ Res Public Health, 2018. **15**(6).

24. Bodeau-Livinec, F., et al., *Neurocognitive testing in West African children 3-6 years of age: Challenges and implications for data analyses.* Brain Research Bulletin, 2018.

25. World Health Organization (WHO). *Physical Status: the use and interpretation of anthropometry. A report of a WHO Expert Committee.* , in *WHO Technical Series, Geneva.* 1995, WHO.

26. World Health Organization (WHO). *The WHO Child Growth Standards*. 2006 [cited 2019.

For peer review only

27. Jenss, R.M. and N. Bayley, *A mathematical method for studying the growth of a child.* Human Biology, 1937. **9**(4): p. 556.

28. Botton, J., et al., *Postnatal weight and height growth modeling and prediction of body mass index as a function of time for the study of growth determinants.* Ann Nutr Metab, 2014. **65**(2-3): p. 156-66.

29. Benjamin, G., *On the nature of the function expressive of the law of human mortality, and ona new mode of determining the value of life contingencies.* . Philosophical Transactions of the Royal Society of London B: Biological Sciences, 1825: p. 513-585.

30. Karkach, A., *Trajectories and models of individual growth.* Demographic Research, 2006. **15**: p. 347-400.

31. Tjørve, K.M.C. and E. Tjørve, *The use of Gompertz models in growth analyses, and new Gompertz-model approach: An addition to the Unified-Richards family.* PLOS ONE, 2017. **12**(6): p. e0178691.

32. Winsor, C.P., *The Gompertz Curve as a Growth Curve.* Proc Natl Acad Sci U S A, 1932. **18**(1): p. 1-8.

33. Berkey, C.S. and R.B. Reed, *A model for describing normal and abnormal growth in early childhood.* Hum Biol, 1987. **59**(6): p. 973-87.

34. Hauspie, R.C., *Mathematical models for the study of individual growth patterns.* Rev Epidemiol Sante Publique, 1989. **37**(5-6): p. 461-76.

35. Team, R.D.C., *R: A language and environment for statistical computing.* . 2017, R Foundation for Statistical Computing: Vienna, Austria.

36. Comets, E., A. Lavenu, and M. Lavielle, *Parameter Estimation in Nonlinear Mixed Effect Models Using saemix, an R Implementation of the SAEM Algorithm.* Journal of Statistical Software, 2017. **80**(3): p. 1-42.

For peer review only

37. World Health Organization (WHO). *The WHO Child Growth Standards*. 2006 [cited 2019 2019 02 February]; Available from: https://www.who.int/childgrowth/standards/en/.

38. Anderson, C., et al., *Comparing predictive abilities of longitudinal child growth models.* Stat Med, 2018.

39. Accrombessi, M., et al., *Malaria in Pregnancy Is a Predictor of Infant Haemoglobin Concentrations during the First Year of Life in Benin, West Africa.* PLoS One, 2015. **10**(6): p. e0129510.

40. Regnault, N., et al., *Comparative Study of Four Growth Models Applied to Weight and Height Growth Data in a Cohort of US Children from Birth to 9 Years.* Annals of Nutrition and Metabolism, 2014. **65**(2-3): p. 167-174.

41. Chirwa, E.D., et al., *Postnatal growth velocity and overweight in early adolescents: a comparison of rural and urban African boys and girls.* Am J Hum Biol, 2014. **26**(5): p. 643-51.

FIGURE LEGENDS

Figure 1 Residuals of weight models for boys and girls

(Data of the first two deciles was gathered, as there were more than 10% of the data at birth)

Figure 2 Residuals of height models for boys and girls

(Data of the first two deciles was gathered, as there were more than 10% of the data at birth)

Figure 3 Weight and height mean growth trajectories from the Jenss-Bayley model. (A) Weight growth curve. (B) Height growth curve. The solid (black) line implies boys curve and dashed line implies girls

Supplementary Figure 1 Population flowchart: Follow-up until 6 years and showing how the final sample has been selected for growth modelling

For peer review only

For peer review only

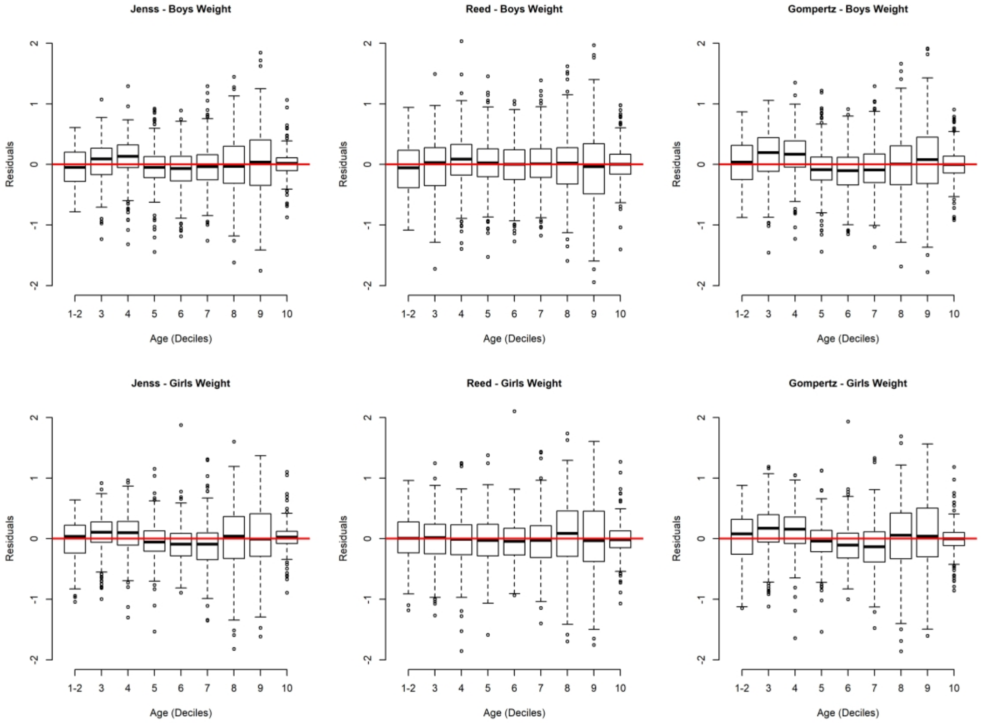

Figure 1 Residuals of weight models for boys and girls
(Data of the first two deciles was gathered, as there were more than 10% of the data at birth)

270x202mm (400 x 400 DPI)

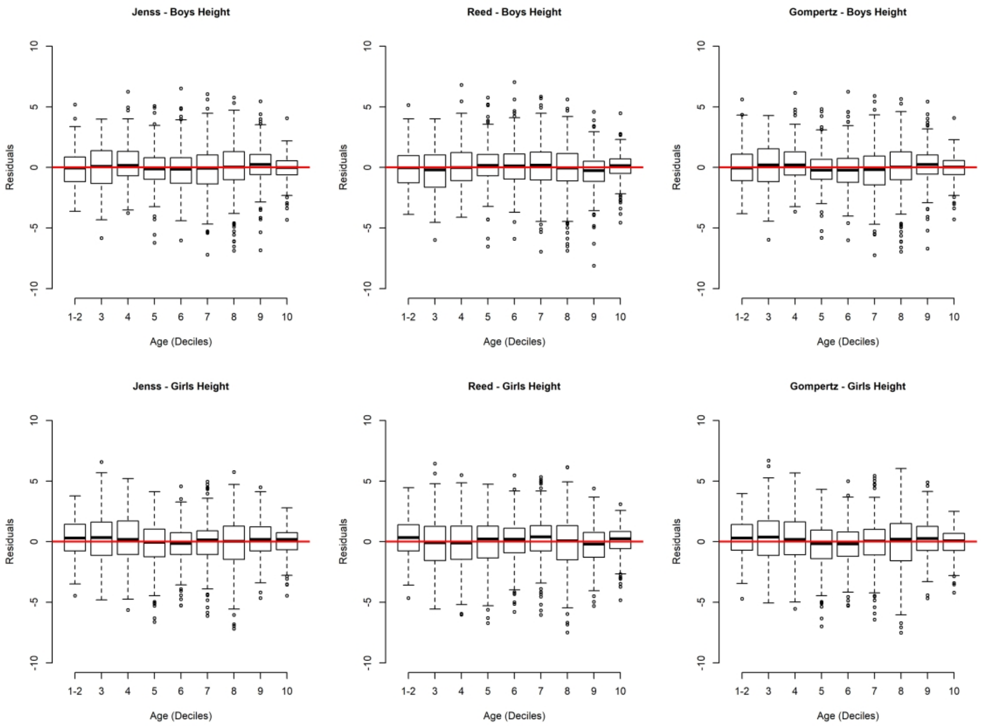

Figure 2 Residuals of height models for boys and girls
(Data of the first two deciles was gathered, as there were more than 10% of the data at birth)

270x202mm (400 x 400 DPI)

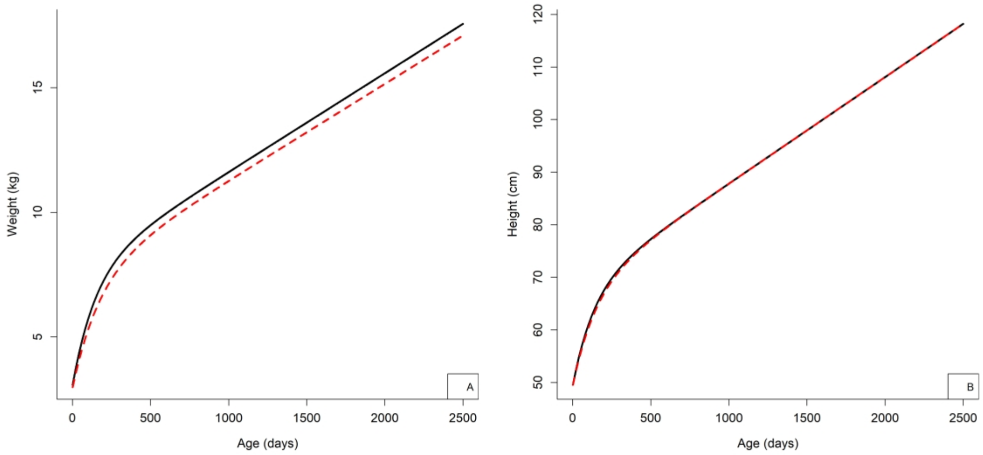

Figure 3 Weight and height mean growth trajectories from the Jenss-Bayley model. (A) Weight growth curve. (B) Height growth curve. The solid (black) line implies boys curve and dashed line implies girls

399x202mm (300 x 300 DPI)

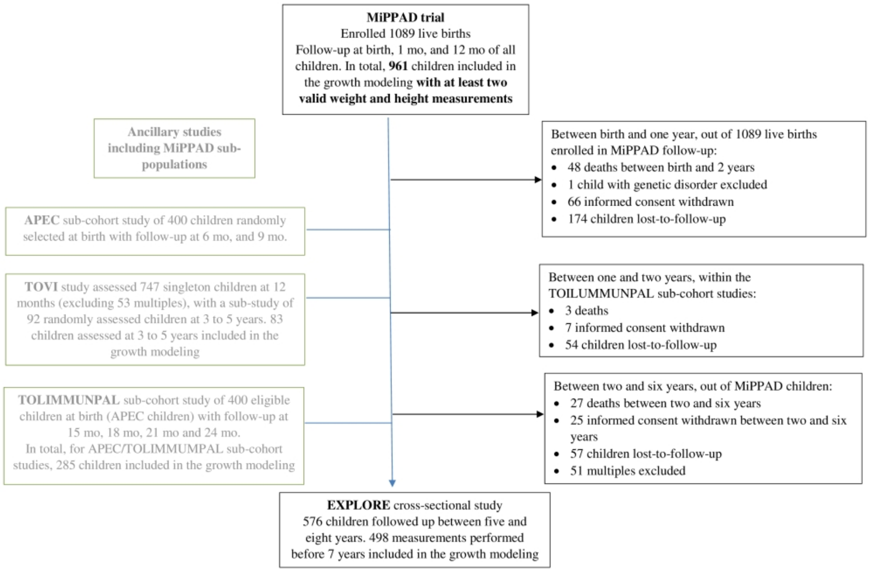

Supplementary Figure 1. Population flowchart: Follow-up until 6 years and showing how the final sample has been selected for growth modelling

297x210mm (300 x 300 DPI)

Supplementary tables

Supplementary Table 1. Number of children with anthropometric measurements at different age intervals. Data from boys (n=461) and girls (n=500) from birth to around six years of age

Age group	Boys	Girls
	n	n
0 – 1 yr	461	499
1 - 2 yr	196	219
2 - 3 yr	97	115
3 - 4 yr	8	10
4 - 5 yr	41	24
5 – 7 yr	253	249

Supplementary Table 2. Summary of maternal and child characteristics in the prospective child cohort in Benin, Sub-Saharan Africa.

	**Boys (n= 461)**		**Girls (n=500)**	
	**n/N**	**%**	**n/N**	**%**
Center				
Allada	51/371	13.8	49/400	12.3
Attogon	83/371	22.4	104/400	26.0
Sékou	237/371	62.9	247/400	61.8
Maternal characteristics				
Mean maternal age at the start of follow up (yr)	451/461	26.0 +/- 5.4	490/500	25.6 +/- 5.5
Maternal education [a]	112/451	24.8	106/490	21.6

For peer review only - http://bmjopen.bmj.com/site/about/guidelines.xhtml

**Supplementary table 2. continued**				
Estimated maternal pre-pregnancy BMI (kg/m^2)				
Underweight (<18.5)	61/373	16.4	67/400	16.8
Normal (18.5 – 24.9)	262/373	70.2	298/400	74.5
Overweight (25-29.9)	35/373	9.4	26/400	6.5
Obese (≥ 30)	15/373	4.02	9/400	2.3
Gravidity				
Multigravida	305/373	81.8	326/400	81.5
Primigravida	68/373	18.2	74/400	18.5
Child characteristics				
Low birth weight (<2.5 kg)	39/440	8.9	66/481	13.7
Preterm	28/443	6.3	33/484	6.8
Malaria at 1 year	35/351	10.0	38/348	10.9
Anemia at 1 year (Hb≤110 g/L)	261/354	73.7	233/348	66.9

[a] Defined as the ability to read or write

For peer review only - http://bmjopen.bmj.com/site/about/guidelines.xhtml

Supplementary Table 3. Parameters estimates for three candidate models [a] fitted to weight and height

Model	Parameters	Boys		Girls	
		Mean	SD	Mean	SD
Weight models					
Jenss					
	A	1.11	0.05	1.08	0.06
	B	5.53	0.15	5.55	0.18
	C	1.52	0.16	1.49	0.15
	D	4.97	0.24	5.15	0.37
Adapted Gompertz	A	2.02	0.08	1.96	0.07
	B	0.90	<0.01	0.85	< 0.01
	C	4.67	0.24	4.85	0.25
	D	5.50	0.13	5.49	0.15
Reed	A	-1.18	<0.01	-0.89	0.12
	B	-5.87	0.18	-5.78	0.18
	C	0.39	0.08	0.28	0.07
	D	1.44	<0.01	1.34	<0.01
Height models					
Jenss					
	A	3.90	0.02	3.90	0.02
	B	3.90	0.08	3.90	0.06
	C	2.90	0.01	2.90	0.06
	D	4.90	0.09	5.00	0.07

For peer review only

Supplementary table 3. continued

Adapted Gompertz	A	4.21	0.02	4.2	0.02
	B	0.31	< 0.01	0.3	<0.01
	C	4.83	0.13	4.9	0.12
	D	3.92	0.07	3.9	0.06
Reed	A	3.50	0.03	3.5	0.03
	B	-4.20	0.09	-4.2	0.08
	C	1.80	0.02	1.7	0.02
	D	2.80	< 0.01	2.8	<0.01

[a] the sign of the parameters for the adapted Gompertz and the Reed model was constrained by using exponential functions, as shown in equation 2 for the Jenss-Bayley model.

Supplementary Table 4. Stunting, underweight and wasting in children using predicted data from the Jenss-Bayley model. n (%)

Sex	Underweight (WAZ<–2)	Stunting (HAZ<-2)	Wasting (WHZ<-2)
Boys (n=461)	161/461 (35)	37/461 (8)	125/451 (27.7)
Girls (n=500)	168/500 (34)	22/500 (4)	190/495 (38.4)

For peer review only

For peer review only - http://bmjopen.bmj.com/site/about/guidelines.xhtml
